# Generalized escape criteria for fractals via convex viscosity approximation iterations

**DOI:** 10.1371/journal.pone.0349186

**Published:** 2026-06-10

**Authors:** Shuai Wang, Khaleel Ahmad, Yining Yang, Luminiţa-Ioana Cotîrlă, Daniel Breaz

**Affiliations:** 1 Department of Mathematics and Physics, Changchun Guanghua University, Changchun, China; 2 Department of Mathematics, University of Management and Technology, Lahore, Pakistan; 3 Center for Theoretical Physics, Khazar University, Baku, Azerbaijan; 4 School of Foreign Studies, Northeastern University at Qinhuangdao, Qinhuangdao, China; 5 Department of Mathematics, Technical University of Cluj-Napoca, Cluj-Napoca, Romania; 6 Department of Mathematics, 1 Decembrie University of Alba Iulia, Romania; Ministry of Education, MOROCCO

## Abstract

In this manuscript, we present the generation of Mandelbrot sets, Julia set fractals, and Biomorphs using a two-step viscosity approximation method applied to the complex function $W(z)=z^{n}+uz+r$, where n≥2 and u,r∈C. The two-step viscosity approximation method is employed to establish new escape criteria for Julia sets, Mandelbrot sets, and Biomorphs. Subsequently, the viscosity approximation process is extended by incorporating *m*-convexity, and the corresponding escape criteria are generalized for these fractals. Further, we introduce the viscosity approximation process with *s*-convexity and develop the associated escape criteria for the same fractal structures. Additionally, we provide a comparative visualization of Mandelbrot sets, Julia set fractals, and Biomorphs using the standard viscosity approximation process, as well as its *m*-convex and *s*-convex variants, applied to the same complex polynomial function. High-resolution fractal images are produced using MATLAB R2024a with 50 iterations and a figure resolution of 800, highlighting the differences in the resulting structures for identical parameter values.

## 1. Introduction

The captivating fractal structures that cover nature’s canvas reveal an amazing and deep intricacy that defies conventional geometry as patterns constantly reproduce themselves at smaller dimensions. Mandelbrot [1] established fractal geometry by showing how recursive mathematical functions might capture the irregularity of natural structures. Zhang et al. [2] suggested a chaos-based image encryption method employing Hilbert curves and H-fractals, so proving the significance of fractals in visual data security. Khalil et al. [3] demonstrated the computational efficiency of fractals in multimedia processing, so contributing to real-time imaging.

Kumar [4] investigated the cryptographic possibilities of fractals. Ouyang et al. [5] created fractal drawings at the junction of mathematics and art that combined visual symmetry with recursive complexity. Krzysztofik [6] provided a complete review of fractals in antennas and metamaterials. Costanzo and Venneri [7] developed polarization-insensitive metamaterials for energy. Harris [8] underlined the way fractal design should be incorporated into architecture. Julia [9] was among the first to investigate the iteration of rational functions. Dang et al. [10] presented hypercomplex iterations and distance estimation for rendering high-dimensional fractals. Wang and Song [11] expanded this by producing Julia sets in bi-complex space and general Mandelbrot sets. Parise and Rochon [12] carried on this path using a study on tricomplex polynomial dynamics, so allowing analysis of fractals in three-complex-dimensional environments. Pickover [13] proposed the idea of biomorphs and generate the biologically inspired fractals.

Kaboudian et al. [14] real-time interactive simulations of chaos and fractals. Jakubska-Busse et al. [15] investigated the effect of non-standard complex numbers in biomorph generation. In “Fractals Everywhere,” Barnsley [16] provided freely available, mathematically exact treatment of fractals. Devaney [17] matched this with providing an iterative function theory and chaotic dynamical system. Kumari et al. [18,19] proposed generalized multivalued iterated function systems and investigated their many structures in b-metric spaces.

Hundertmark-Zaušková [20] showed by applying fixed-point iterations to moving geometries in fluid-structure interactions. Rahmani et al. [21] used fixed-point techniques for travel time estimate from sparse floating car data. Strogatz [22] reinforced the importance of convergence and iteration by offering a conceptual yet exact treatment of nonlinear dynamics and chaos. Usurelu et al. [23] combined numerical iteration with visual creativity. Mann [24] introduced the mean value iteration technique. Halpern [25] studied fixed points of non-expansive mappings. Nandal and Chugh [26] studied zero of accretive operators. Nandal et al. [27] investigated on variational inequalities involving pseudo-contractive operators. Postolache et al. [28] showed strong convergence results. Nandal et al. [29] proposed iterative schemes for maximal monotone operators.

Zou et al. [30] investigated the Mandelbrot and Julia sets using Picard-Mann iteration. Shahid et al. [31] expanded this by including s-convexity. Chauhan et al. [32] used Ishikawa iteration and generate the Julia sets fractals.

Saleem et al. [33] generalized the fractals in neutrosophic metric spaces. Kwun et al. [34] used Jungck-CR iteration with s-convexity and generated fractal. Kumari, and Chugh [35] introduced a new class of fractals by using the SP orbits and s-convexity. Jolaoso and Khan [36] proposed generalized escape time algorithms. Tassaddiq [37] used Jungck-Noor orbit and presented an extended escape criterion that improved control over fractal divergence patterns. Bhoria et. al [38] generated the Julia and Mandelbrot set fractals by using Picard Thakor iteration. Gdawiec, et al. [39] Generalized Logistic maps in the complex plane. Tassaddique et al. [40] studied fractal generation through fixed-point iteration processes that are modified. They were able to show that classical iteration schemes can be substantially enriched by changing them. The use of new iterative schemes has been also developed by Tanveer and Gdawiec [41], who applied the CR iteration scheme to create Mandelbrot sets related to the function $z^{p}+\text{log}c^{t}$. Tassaddiq et al. [42] studied the Mann-iterative scheme in the framework of h-convexity of fractal generation. They introduced the application of convexity ideas to the iteration theory to give an analytical context that is generalized and guarantees convergence, and at the same time gives sets that are rich in fractal patterns. The escape conditions were also developed by Tassaddiq et al. [43] in the generation of fractals through the Picard Thakur hybrid iteration. Not only were their findings more efficient in computational efficiency. Tanveer, et. al [44] used Mann and Picard Mann iterations to analyze the Mandelbrot sets of $z^{p}+\text{log}c^{t}$. They compared their study to show the influence of varying iteration schemes on the convergence rate, escape behavior and fractal geometry.

The Hybrid Picard-S iteration scheme was presented in Tomar et al. [45] to conduct the detailed analysis of the escape of complex fractals. Generation of fractal using transcendental functions have also been extended by Alam et al. [46] who suggested a successful iterative process involving complex sine functions generate Julia and Mandelbrot sets. A new line of study connecting number theory with fractal visualization was introduced by Sharma et al. [47], who explored the application of the Fibonacci sequence in the development and visualization of fractals. Previous underlying literature by Tomar et al. [48] considered Julia and Mandelbrot sets that occurred when considering complex cosine functions by applying fixed-point iterative schemes. Alam et al. [49], who also developed escape conditions on orbits based on s-convexity and explored behavioral change in Mandelbrot and Julia sets. Their work presents a coherent mathematical structure of the analysis of divergence of orbits and transitions of stability in complex iterative systems. The dynamic of whole transcendental functions was studied by Prajapati et al. [50], who did a systematic study of Julia sets created using Mann-type schemes. Variants of Mandelbrot and Julia fractals of higher-order complex polynomial were studied by Tomar et al. [51]. Özdemir et. al [ 52–56] discussed the *m*-convexity, and *s*-convexity.

➢ In section 2, we discuss Julia set, Mandelbrot set, Mann iteration method, Halpern method, and viscosity approximation techniques, m-convexity, and s-convexity from existing literature.
➢ In section 3, we discuss escape criteria Julia, Mandelbrot sets and Biomorph by using viscosity approximation process, viscosity approximation process with *m*-convexity, and viscosity approximation process with *s*-convexity.
➢ In section 4, we show the algorithms (viscosity approximation method, viscosity approximation process with m-convexity, and viscosity approximation process with s-convexity for Julia set fractals and generate the Julia set fractals by using MATLAB R2024a.
➢ In section 5, we discuss the algorithms for Mandelbrot set fractals via viscosity approximation method, viscosity approximation process with m-convexity, and viscosity approximation process with s-convexity. We generate the Mandelbrot set fractals by using MATLAB R2024a.
➢ In section 6, we demonstration the algorithms for Biomorph by using viscosity approximation method, viscosity approximation process with m-convexity, and viscosity approximation process with s-convexity.
➢ In section 7, the ANI, Time plot, AET, and NAI are deliberated for viscosity approximation method, viscosity approximation process with m-convexity, and viscosity approximation process with s-convexity.
➢ In section 8, we discuss the comparison between viscosity approximation method, viscosity approximation process with m-convexity, and viscosity approximation process with s-convexity.
➢ In section 9, we derive conclusions from our results and future work.

The main objectives of this study are threefold. First, we establish new escape criteria for Julia sets, Mandelbrot sets, and Biomorphs generated by the complex polynomial $W(z)=z^{n}+uz+r,$ where n≥2 and u,r∈C using a two-step viscosity approximation method. Second, we extend the viscosity approximation process by incorporating *m*-convexity and *s*-convexity, and derive the corresponding escape criteria for these fractal structures. Third, we provide a comparative visualization and quantitative analysis (including Average Escape Time and Non-escaping Area Index) of the resulting fractals under the standard viscosity approximation and its *m*-convex and *s*-convex variants, using high-resolution images generated in MATLAB R2024a. Through this systematic investigation, we aim to demonstrate how the choice of convexity affects boundary sharpness, symmetry, complexity, and computational efficiency.

## 2. Preliminaries

In this section, we discuss some fundamental definitions including Julia, Mandelbrot sets and Biomorph from existing literature. We denote C is a set of complex numbers and R is denoted for set of real numbers.

**Definition 2.1:** [9] The filled Julia set $F_{t_{r}}$ of the complex function $t_{r}:C\to C,$ where r∈C, is the set of points in the complex plan whose orbits under $t_{r}$ are bounded, i.e.,


$$\begin{array}{c} F_{t_{r}}=\left\{z\in C:\overset{\infty }{\underset{j=\text{0}}{\left\{\left|\overset{j}{\underset{r}{t}}(z)\right|\right\}}}\text{ is bounded}\right\}, \end{array}$$ (1)

where $\overset{j}{\underset{r}{t}}$ denotes the jth iteration of the function $t_{r}.$ The Julia set $J_{t_{r}}$ of $t_{r}$ is the boundary of the filled Julia set $F_{t_{r}},$, i.e., $J_{t_{r}}=\partial F_{t_{r}}.$

Benoit B. Mandelbrot [1], a French mathematician, helped Julia.

**Definition 2.2:** [1] The Mandelbrot set $M_{t_{r}}$ of a function $t_{r}:C\to C,$ is defined as the collection of all numbers r∈C for which the filled Julia set $F_{t_{r}},$ remains connected, i.e.,


$$\begin{array}{c} M_{t_{r}}=\left\{r\in C:F_{t_{r}}\text{ is connected}\right\}. \end{array}$$ (2)

**Definition 2.3:** [24] Assume that *D* is a subset of a Banach space that is closed and convex. A self-mapping *W* is a non-expansive mapping in *D*. If a sequence $\left\{z_{n}\right\}$ satisfied the below pattern, then it is said to be Mann iteration process:


$$\begin{array}{c} z_{n+\text{1}}=\left(\text{1}-\alpha_{n}\right)z_{n}+\alpha_{n}W\left(z_{n}\right),\;n\geq \text{0}, \end{array}$$ (3)

where $z_{\text{0}}$ is any starting point in *D* and a sequence $\alpha_{n}$ in (0,1).

**Definition 2.4:** [25] Suppose that $\left\{z_{n}\right\}$ is a sequence in C and $z_{\text{0}}\in C$ is an initial point. Then viscosity approximation method is defined as:


$$\begin{array}{c} z_{n+\text{1}}=\alpha_{n}g\left(z_{n}\right)+\left(\text{1}-\alpha_{n}\right)W\left(z_{n}\right), \end{array}$$ (4)

where, g:C→C be a mapping and $\alpha_{n}$ in (0,1).

**Definition 2.5:** [51] Assume that I⊆R and a function f:[0,b]→R, where b∈I, is said to be m-convex if satisfied the following inequality, for x,y∈[0,b]:


$$\begin{array}{c} f\left(tx+m(\text{1}-t)y\right)\leq tf(x)+m(\text{1}-t)f(y), \end{array}$$

wherem, t∈[0,1].

**Definition 2.6:** [52] A function f:[0, ∞)→[0, ∞) is called first sense s-convex if it satisfied the below inequality, forx, y∈[0,∞):


$$\begin{array}{c} f\left(tx+m(\text{1}-t)y\right)\leq t^{s}f(x)+(\text{1}-t{)}^{s}f(y), \end{array}$$ (5)

where t∈[0,1], ands∈(0,1].

Pickover [13] introduced the Biomorph in 1986. In which following modification was creating Julia set that are given below:


$$\begin{array}{c} \left|Re(z)\right|<R\text{ or }\left|Im(z)\right|<R, \end{array}$$

where, *R* is denoted as escape radius. The Re(z) and Im(z) are real and Imaginary part of a complex number z∈C.

## 3. Escape criterion

In this section, we will discuss the escape criterion for Julia set, Mandelbrot set, and Biomorphs for viscosity approximation method, viscosity approximation method with *m*-convexity, and viscosity approximation method with *s*-convexity.

### 3.1. Escape criterion for Viscosity Approximation method

In this section, we discuss the escape criterion for Julia and Mandelbrot set fractals. We use $\alpha =\underset{n\in N}{\text{inf}}\alpha_{n}\;$ where α∈(0,1) throughout the paper. We generalized the results [25], and generate Julia and Mandelbrot set fractals. The viscosity approximation process for any initial point $z_{\text{0}}\in C$ can be stated in the complex space as:


$$\begin{array}{c} \left\{ \begin{array}{c} y_{j}=\alpha g\left(z_{j}\right)+(\text{1}-\alpha )W\left(z_{j}\right),\\ z_{j+\text{1}}=\;W\left(y_{j}\right)\; \end{array}\right.\; \end{array}$$ (6)

for all j≥0, where α∈(0,1) and the functions W,g:C→C are self-mappings on C This two-step viscosity approximation iteration process has better control as compare to Picard or Mann iterations. The escape criterion is important in the visualization of Mandelbrot and Julia set fractals. we discuss an escape criterion for the following complex function that is defined as:


$$\begin{array}{c} W(z)=z^{n}+uz+r, \end{array}$$ (7)

where n≥2 and u,r∈C, by using viscosity approximation method given in (6). Let g(z)=az+b be a complex contraction with a,b∈C and |a|<1, we have −∣a∣>−1. Then by use −a≥−∣a∣, it follows that −a>−1, and hence−a−1<0.

**Theorem 3.1.1:** Suppose $\left|z_{\text{0}}\right|\geq \text{max}\left\{|r|,|b|,\;{\left(\text{2}+|u|\right)}^{\frac{\text{1}}{n-\text{1}}},\;{\left(\frac{\text{2}+\alpha +(\text{1}-\alpha )|u|}{\text{1}-\alpha }\right)}^{\frac{\text{1}}{n-\text{1}}}\right\},$ where α∈(0,1) and u,r,a,b∈C, and |a|<1. If the sequence ${\left\{z_{j}\right\}}_{N\cup \left\{\text{0}\right\}}$ satisfied the viscosity approximation iteration process that is given in (6). Then$\left|z_{j}\right|\to \infty \text{ as }j\to \infty .$

**Proof:** From (6), consider


$$\begin{array}{cc} \left|y_{\text{0}}\right| & =\left|\alpha g\left(z_{\text{0}}\right)+(\text{1}-\alpha )W\left(z_{\text{0}}\right)\right|\\ & =\left|\alpha \left(az_{\text{0}}+b\right)+(\text{1}-\alpha )\left(\overset{n}{\underset{\text{0}}{z}}+uz_{\text{0}}+r\right)\right|\\ & =\left|(\text{1}-\alpha )\overset{n}{\underset{\text{0}}{z}}+(\text{1}-\alpha )uz_{\text{0}}+(\text{1}-\alpha )r+\alpha az_{\text{0}}+\alpha b\right|\\ & \geq \left|(\text{1}-\alpha )\overset{n}{\underset{\text{0}}{z}}+(\text{1}-\alpha )uz_{\text{0}}+(\text{1}-\alpha )r+\alpha az_{\text{0}}\right|-|\alpha b|\\ & \geq \left|(\text{1}-\alpha )\overset{n}{\underset{\text{0}}{z}}+(\text{1}-\alpha )uz_{\text{0}}+\alpha az_{\text{0}}\right|-\left|(\text{1}-\alpha )r\right|-|\alpha b|. \end{array}$$

The assumption $\left|z_{\text{0}}\right|\geq \text{max}\left\{|r|,|b|,\;{\left(\text{2}+|u|\right)}^{\frac{\text{1}}{n-\text{1}}},\;{\left(\frac{\text{2}+\alpha |a|+(\text{1}-\alpha )|u|}{\text{1}-\alpha }\right)}^{\frac{\text{1}}{n-\text{1}}}\right\},$ implies $-|r|\geq -\left|z_{\text{0}}\right|\;$ and $-|b|\geq -\left|z_{\text{0}}\right|,$ then we obtain


$$\begin{array}{cc} \left|y_{\text{0}}\right| & \geq (\text{1}-\alpha )\left|\overset{n}{\underset{\text{0}}{z}}\right|-(\text{1}-\alpha )|u|\left|z_{\text{0}}\right|-\alpha |a|\left|z_{\text{0}}\right|-\left|(\text{1}-\alpha )z_{\text{0}}\right|-\alpha \left|z_{\text{0}}\right|\\ & \geq (\text{1}-\alpha )\left|\overset{n}{\underset{\text{0}}{z}}\right|-(\text{1}-\alpha )|u|\left|z_{\text{0}}\right|-\alpha |a|\left|z_{\text{0}}\right|-\left|z_{\text{0}}\right|\\ & =\left|z_{\text{0}}\right|\left((\text{1}-\alpha )\left|\overset{n-\text{1}}{\underset{\text{0}}{z}}\right|-(\text{1}-\alpha )|u|-\alpha |a|-\text{1}\right). \end{array}$$

We assume that |a|<1, we have


$$\begin{array}{c} \geq \left|z_{\text{0}}\right|\left((\text{1}-\alpha )\left|\overset{n-\text{1}}{\underset{\text{0}}{z}}\right|-(\text{1}-\alpha )|u|-\alpha -\text{1}\right). \end{array}$$

The condition $\left|z_{\text{0}}\right|>{\left(\frac{\text{2}+\alpha +|u|(\text{1}-\alpha )}{\text{1}-\alpha }\right)}^{\frac{\text{1}}{n-\text{1}}},$ so $\left|y_{\text{0}}\right|>\left|z_{\text{0}}\right|.$ Therefore, the second step of viscosity approximation method is:


$$\begin{array}{c} \left|z_{\text{1}}\right|=\left|W\left(y_{\text{0}}\right)\right|=\left|\overset{n}{\underset{\text{0}}{y}}+uy_{\text{0}}+r\right|\geq \left|\overset{n}{\underset{\text{0}}{y}}\right|-|u|\left|y_{\text{0}}\right|-|r|. \end{array}$$

Since, $\left|z_{\text{0}}\right|\geq \text{max}\left\{|r|,|b|,\;{\left(\text{2}+|u|\right)}^{\frac{\text{1}}{n-\text{1}}},\;{\left(\frac{\text{2}+\alpha +(\text{1}-\alpha )|u|}{\text{1}-\alpha }\right)}^{\frac{\text{1}}{n-\text{1}}}\right\},$ implies $-|r|\geq -\left|z_{\text{0}}\right|,$ and $\left|y_{\text{0}}\right|\geq \left|z_{\text{0}}\right|.$ We obtain,


$$\begin{array}{c} \left|z_{\text{1}}\right|\geq \left|\overset{n}{\underset{\text{0}}{z}}\right|-|u|\left|z_{\text{0}}\right|-\left|z_{\text{0}}\right|=\left|z_{\text{0}}\right|\left(\left|\overset{n-\text{1}}{\underset{\text{0}}{z}}\right|-|u|-\text{1}\right). \end{array}$$

Since, $\left|z_{\text{0}}\right|\geq \text{max}\left\{|r|,|b|,\;{\left(\text{2}+|u|\right)}^{\frac{\text{1}}{n-\text{1}}},\;{\left(\frac{\text{2}+\alpha +(\text{1}-\alpha )|u|}{\text{1}-\alpha }\right)}^{\frac{\text{1}}{n-\text{1}}}\right\},$ then$\left|z_{\text{1}}\right|\geq \left|z_{\text{0}}\right|.$

Thus, there exists a real number σ>0 such that$\;\left|z_{\text{1}}\right|>(\sigma +\text{1})\left|z_{\text{0}}\right|.$ Continuing this process we have


$$\begin{array}{cc} \left|z_{\text{2}}\right|>(\sigma & +\text{1}{)}^{\text{2}}\left|z_{\text{0}}\right|,\\ & \vdots \end{array}$$

$$\begin{array}{c} \left|z_{j}\right|>(\sigma +\text{1}{)}^{j}\left|z_{\text{0}}\right|. \end{array}$$

Hence, $\left|z_{j}\right|\to \infty \text{ as }j\to \infty .$

We obtain the following corollary as a refinement of Theorem 3.1.1.

**Corollary 3.1.1:** Assume that $\;\{z_{j}\}$ is a sequence defined in (6) and let


$$\begin{array}{c} \left|z_{k}\right|>\text{max}\left\{|r|,|b|,\;{\left(\text{2}+|u|\right)}^{\frac{\text{1}}{n-\text{1}}},\;{\left(\frac{\text{2}+\alpha +(\text{1}-\alpha )|u|}{\text{1}-\alpha }\right)}^{\frac{\text{1}}{n-\text{1}}}\right\} \end{array}$$

for some k≥0. Then, there exists σ>0 such that $\left|z_{k+j}\right|>(\text{1}+\sigma {)}^{j}\left|z_{k}\right|$ and we have $\left|z_{j}\right|\to \infty \text{ as }j\to \infty .$

### 3.2 Escape criterion for Viscosity Approximation method with m-convexity

In this section, we generalized the result of section 3.1, with m-convexity. We discuss the escape criterion for Julia and Mandelbrot set fractals for viscosity approximation process with m-convexity where m∈[0, 1].

**Definition 3.2.1:** Assume that W,g:C→C are self-mappings on a complex set C. The viscosity approximation process with m-convexity is define as:


$$\begin{array}{c} \left\{ \begin{array}{c} y_{j}=\alpha g\left(z_{j}\right)+m(\text{1}-\alpha )W\left(z_{j}\right),\\ z_{j+\text{1}}=\;W\left(y_{j}\right)\; \end{array}\right.\; \end{array}$$ (8)

for all j≥0, where α∈(0,1), andm∈[0, 1].

We discuss an escape criterion for the complex function that is defined (7) by using viscosity approximation method with m-convexity given in (8). Let g(z)=az+b be a complex contraction with a,b∈C and |a|<1.

**Theorem 3.2.1:** Suppose $\left|z_{\text{0}}\right|\geq \text{max}\left\{|r|,|b|,\;{\left(\text{2}+|u|\right)}^{\frac{\text{1}}{n-\text{1}}},\;{\left(\frac{\text{1}+\text{2}\alpha }{\left|m|(\text{1}-\alpha \right)}+|u|+\text{1}\right)}^{\frac{\text{1}}{n-\text{1}}}\right\},$ where α∈(0,1) and u,r,a,b∈C, and |a|<1. If the sequence ${\left\{z_{j}\right\}}_{N\cup \left\{\text{0}\right\}}$ satisfied the viscosity approximation iteration process that is given in (8). Then$\left|z_{j}\right|\to \infty \text{ as }j\to \infty .$

**Proof:** The assumption $\left|z_{\text{0}}\right|\geq \text{max}\left\{|r|,|b|,\;{\left(\text{2}+|u|\right)}^{\frac{\text{1}}{n-\text{1}}},\;{\left(\frac{\text{1}+\text{2}\alpha }{\left|m|(\text{1}-\alpha \right)}+|u|+\text{1}\right)}^{\frac{\text{1}}{n-\text{1}}}\right\},$ implies $-|r|\geq -\left|z_{\text{0}}\right|\;$and$-|b|\geq -\left|z_{\text{0}}\right|,$ and from (8), we have,


$$\begin{array}{cc} \left|y_{\text{0}}\right| & =\left|\alpha g\left(z_{\text{0}}\right)+m(\text{1}-\alpha )W\left(z_{\text{0}}\right)\right|\\ & =\left|\alpha \left(az_{\text{0}}+b\right)+m(\text{1}-\alpha )\left(\overset{n}{\underset{\text{0}}{z}}+uz_{\text{0}}+r\right)\right|\\ & =\left|m(\text{1}-\alpha )\left(\overset{n}{\underset{\text{0}}{z}}+uz_{\text{0}}+r\right)+\alpha az_{\text{0}}+\alpha b\right|\\ & \geq |m|(\text{1}-\alpha )\left({\left|z_{\text{0}}\right|}^{n}-|u|\left|z_{\text{0}}\right|-\left|z_{\text{0}}\right|\right)-\alpha |a|\left|z_{\text{0}}\right|-\alpha \left|z_{\text{0}}\right|\\ & \geq \left|z_{\text{0}}\right|\left(|m|(\text{1}-\alpha )\left({\left|z_{\text{0}}\right|}^{n-\text{1}}-|u|-\text{1}\right)-\alpha |a|-\alpha \right). \end{array}$$

We assume that |a|<1, so


$$\begin{array}{c} \geq \left|z_{\text{0}}\right|\left(|m|(\text{1}-\alpha )\left({\left|z_{\text{0}}\right|}^{n-\text{1}}-|u|-\text{1}\right)-\text{2}\alpha \right). \end{array}$$

Since, $\left|z_{\text{0}}\right|\geq \text{max}\left\{|r|,|b|,\;{\left(\text{2}+|u|\right)}^{\frac{\text{1}}{n-\text{1}}},\;{\left(\frac{\text{1}+\text{2}\alpha }{\left|m|(\text{1}-\alpha \right)}+|u|+\text{1}\right)}^{\frac{\text{1}}{n-\text{1}}}\right\},$ so $\left|y_{\text{0}}\right|>\left|z_{\text{0}}\right|.$ Therefore, the second step of viscosityapproximation method is:


$$\begin{array}{c} \left|z_{\text{1}}\right|=\left|W\left(y_{\text{0}}\right)\right|=\left|\overset{n}{\underset{\text{0}}{y}}+uy_{\text{0}}+r\right|\geq \left|\overset{n}{\underset{\text{0}}{y}}\right|-|u|\left|y_{\text{0}}\right|-|r|. \end{array}$$

Since, $\left|z_{\text{0}}\right|\geq \text{max}\left\{|r|,|b|,\;{\left(\text{2}+|u|\right)}^{\frac{\text{1}}{n-\text{1}}},\;{\left(\frac{\text{1}+\text{2}\alpha }{\left|m|(\text{1}-\alpha \right)}+|u|+\text{1}\right)}^{\frac{\text{1}}{n-\text{1}}}\right\},$ implies $-|r|\geq -\left|z_{\text{0}}\right|,$ and $\left|y_{\text{0}}\right|\geq \left|z_{\text{0}}\right|.$ We obtain,


$$\begin{array}{c} \left|z_{\text{1}}\right|\geq \left|\overset{n}{\underset{\text{0}}{z}}\right|-|u|\left|z_{\text{0}}\right|-\left|z_{\text{0}}\right|=\left|z_{\text{0}}\right|\left(\left|\overset{n-\text{1}}{\underset{\text{0}}{z}}\right|-|u|-\text{1}\right) \end{array}$$

Since, $\left|z_{\text{0}}\right|\geq \text{max}\left\{|r|,|b|,\;{\left(\text{2}+|u|\right)}^{\frac{\text{1}}{n-\text{1}}},\;{\left(\frac{\text{1}+\text{2}\alpha }{\left|m|(\text{1}-\alpha \right)}+|u|+\text{1}\right)}^{\frac{\text{1}}{n-\text{1}}}\right\},$ then $\left|z_{\text{1}}\right|\geq \left|z_{\text{0}}\right|.$ Thus, there exists a real number σ>0such that$\;\left|z_{\text{1}}\right|>(\sigma +\text{1})\left|z_{\text{0}}\right|.$ Continuing this process we have


$$\begin{array}{cc} \left|z_{\text{2}}\right|>(\sigma & +\text{1}{)}^{\text{2}}\left|z_{\text{0}}\right|,\\ & \vdots \end{array}$$

$$\begin{array}{c} \left|z_{j}\right|>(\sigma +\text{1}{)}^{j}\left|z_{\text{0}}\right|. \end{array}$$

Hence, $\left|z_{j}\right|\to \infty \text{ as }j\to \infty .$

We obtain the following corollary as a refinement of Theorem 3.2.1.

**Corollary 3.2.1:** Assume that $\;\{z_{j}\}$ is a sequence define in (8) and let


$$\begin{array}{c} \left|z_{k}\right|>\text{max}\left\{|r|,|b|,\;{\left(\text{2}+|u|\right)}^{\frac{\text{1}}{n-\text{1}}},\;{\left(\frac{\text{1}+\text{2}\alpha }{\left|m|(\text{1}-\alpha \right)}+|u|+\text{1}\right)}^{\frac{\text{1}}{n-\text{1}}}\right\} \end{array}$$

for some k≥0. Then, there exists σ>0 such that $\left|z_{k+j}\right|>(\text{1}+\sigma {)}^{j}\left|z_{k}\right|$ and we have $\left|z_{j}\right|\to \infty \text{ as }j\to \infty .$

**Remark 3.2.1:** In Theorem 3.2.1, if we take the value of *m* is 1. Then it same as Theorem 3.1.1.

### 3.3. Escape criterion for Viscosity Approximation method with s-convexity

In this section, we generalized our results of section 3.1, with s-convexity. We discuss the escape criterion for Julia and Mandelbrot set fractals for viscosity approximation process with s-convexity where s∈(0, 1].

**Definition 3.3.1:** Assume that W,g:C→C are self-mappings on a complex set C. The viscosity approximation process with s-convexity is define as:


$$\begin{array}{c} \left\{ \begin{array}{c} y_{j}=\alpha^{s}g\left(z_{j}\right)+(\text{1}-\alpha {)}^{s}W\left(z_{j}\right),\\ z_{j+\text{1}}=\;W\left(y_{j}\right)\; \end{array}\right.\; \end{array}$$ (9)

for all j≥0, where α∈(0,1), ands∈(0, 1].

The escape criterion is important in the visualization of Mandelbrot and Julia set fractals. we discuss an escape criterion for the complex function that is defined in (7) by using viscosity approximation method with s-convexity given in (9). Let g(z)=az+b be a complex contraction with a,b∈C and |a|<1.

**Theorem 3.3.1:** Suppose $\left|z_{\text{0}}\right|\geq \text{max}\left\{|r|,|b|,\;{\left(\text{2}+|u|\right)}^{\frac{\text{1}}{n-\text{1}}},\;{\left(\frac{\text{2}+s\alpha }{\left(\text{1}-s\alpha \right)}+|u|\right)}^{\frac{\text{1}}{n-\text{1}}}\right\},$ where α∈(0,1) and u,r,a,b∈C,and |a|<1. If the sequence ${\left\{z_{j}\right\}}_{N\cup \left\{\text{0}\right\}}$ satisfied the viscosity approximation iteration process that is given in (9). Then$\left|z_{j}\right|\to \infty \text{ as }j\to \infty .$

**Proof:** The assumption $\left|z_{\text{0}}\right|\geq \text{max}\left\{|r|,|b|,\;{\left(\text{2}+|u|\right)}^{\frac{\text{1}}{n-\text{1}}},\;{\left(\frac{\text{2}+s\alpha }{\left(\text{1}-s\alpha \right)}+|u|\right)}^{\frac{\text{1}}{n-\text{1}}}\right\},$ implies $-|r|\geq -\left|z_{\text{0}}\right|\;$and$-|b|\geq -\left|z_{\text{0}}\right|,$ and from (9), we have,


$$\begin{array}{cc} \left|y_{\text{0}}\right| & =\left|\alpha^{s}g\left(z_{\text{0}}\right)+(\text{1}-\alpha {)}^{s}W\left(z_{\text{0}}\right)\right|\\ & =\left|\alpha^{s}\left(az_{\text{0}}+b\right)+(\text{1}-\alpha {)}^{s}\left(\overset{n}{\underset{\text{0}}{z}}+uz_{\text{0}}+r\right)\right|\\ & =\left|(\text{1}-\alpha {)}^{s}\left(\overset{n}{\underset{\text{0}}{z}}+uz_{\text{0}}+r\right)+\alpha^{s}az_{\text{0}}+\alpha^{s}b\right|\\ & \geq (\text{1}-\alpha {)}^{s}\left({\left|z_{\text{0}}\right|}^{n}-|u|\left|z_{\text{0}}\right|-\left|z_{\text{0}}\right|\right)-\alpha^{s}|a|\left|z_{\text{0}}\right|-\alpha^{s}\left|z_{\text{0}}\right|\\ & \geq (\text{1}-s\alpha )\left|\overset{n}{\underset{\text{0}}{z}}\right|-(\text{1}-s\alpha )|u|\left|z_{\text{0}}\right|-(\text{1}-s\alpha )\left|z_{\text{0}}\right|-s\alpha |a|\left|z_{\text{0}}\right|-s\alpha \left|z_{\text{0}}\right|. \end{array}$$

We assume that |a|<1, then we have


$$\begin{array}{c} \geq (\text{1}-s\alpha )\left|\overset{n}{\underset{\text{0}}{z}}\right|-(\text{1}-s\alpha )|u|\left|z_{\text{0}}\right|-(\text{1}-s\alpha )\left|z_{\text{0}}\right|-s\alpha \left|z_{\text{0}}\right|-s\alpha \left|z_{\text{0}}\right|\geq \left|z_{\text{0}}\right|\left((\text{1}-\alpha )\left(\left|\overset{n-\text{1}}{\underset{\text{0}}{z}}\right|-|u|\right)-s\alpha -\text{1}\right). \end{array}$$

Since, $\left|z_{\text{0}}\right|\geq \text{max}\left\{|r|,|b|,\;{\left(\text{2}+|u|\right)}^{\frac{\text{1}}{n-\text{1}}},\;{\left(\frac{\text{2}+s\alpha }{\left(\text{1}-s\alpha \right)}+|u|\right)}^{\frac{\text{1}}{n-\text{1}}}\right\},$ so $\left|y_{\text{0}}\right|>\left|z_{\text{0}}\right|.$ Therefore, the second step of viscosity approximation method is:


$$\begin{array}{c} \left|z_{\text{1}}\right|=\left|W\left(y_{\text{0}}\right)\right|=\left|\overset{n}{\underset{\text{0}}{y}}+uy_{\text{0}}+r\right|\geq \left|\overset{n}{\underset{\text{0}}{y}}\right|-|u|\left|y_{\text{0}}\right|-|r|. \end{array}$$

Since, $\left|z_{\text{0}}\right|\geq \text{max}\left\{|r|,|b|,\;{\left(\text{2}+|u|\right)}^{\frac{\text{1}}{n-\text{1}}},\;{\left(\frac{\text{2}+s\alpha }{\left(\text{1}-s\alpha \right)}+|u|\right)}^{\frac{\text{1}}{n-\text{1}}}\right\},$ implies $-|r|\geq -\left|z_{\text{0}}\right|,$ and $\left|y_{\text{0}}\right|\geq \left|z_{\text{0}}\right|.$ We obtain,


$$\begin{array}{c} \left|z_{\text{1}}\right|\geq \left|\overset{n}{\underset{\text{0}}{z}}\right|-|u|\left|z_{\text{0}}\right|-\left|z_{\text{0}}\right|=\left|z_{\text{0}}\right|\left(\left|\overset{n-\text{1}}{\underset{\text{0}}{z}}\right|-|u|-\text{1}\right) \end{array}$$

Since, $\left|z_{\text{0}}\right|\geq \text{max}\left\{|r|,|b|,\;{\left(\text{2}+|u|\right)}^{\frac{\text{1}}{n-\text{1}}},\;{\left(\frac{\text{2}+s\alpha }{\left(\text{1}-s\alpha \right)}+|u|\right)}^{\frac{\text{1}}{n-\text{1}}}\right\},$ then $\left|z_{\text{1}}\right|\geq \left|z_{\text{0}}\right|.$ Thus, there exists a real number σ>0 such that $\;\left|z_{\text{1}}\right|>(\sigma +\text{1})\left|z_{\text{0}}\right|.$ Continuing this process we have


$$\begin{array}{cc} \left|z_{\text{2}}\right|>(\sigma & +\text{1}{)}^{\text{2}}\left|z_{\text{0}}\right|,\\ & \vdots \end{array}$$

$$\begin{array}{c} \left|z_{j}\right|>(\sigma +\text{1}{)}^{j}\left|z_{\text{0}}\right|. \end{array}$$

Hence, $\left|z_{j}\right|\to \infty \text{ as }j\to \infty .$

We obtain the following corollaries as a refinement of Theorem 3.3.1.

**Corollary 3.3.1:** Suppose that $\;\{z_{j}\}$ is a sequence define in (6) and let


$$\begin{array}{c} \left|z_{k}\right|>\text{max}\left\{|r|,|b|,\;{\left(\text{2}+|u|\right)}^{\frac{\text{1}}{n-\text{1}}},\;{\left(\frac{\text{2}+s\alpha }{\left(\text{1}-s\alpha \right)}+|u|\right)}^{\frac{\text{1}}{n-\text{1}}}\right\} \end{array}$$

for some k≥0. Then, there exists σ>0 such that $\left|z_{k+j}\right|>(\text{1}+\sigma {)}^{j}\left|z_{k}\right|$ and we have $\left|z_{j}\right|\to \infty \text{ as }j\to \infty .$

**Remark 3.3.1:** In Theorem 3.3.1, if we take the value of *s* is 1. Then it same as Theorem 3.1.1.

## 4. Algorithms for Julia set Fractals

In this section, we discuss the algorithms of Julia sets fractals for complex polynomial $W(z)=z^{n}+uz+r$ where n≥2 and m,r∈C.

Algorithm 1. Julia set generation for Viscosity approximation process

Input: $W\left(z\right)=z^{n}+uz+r$ where n≥2 𝐚𝐧𝐝 u,r∈C. A ⊂ ℂ-area; K-the maximum number of iterations; α∈(0,1)-parameters for the viscosity approximation iterative method; g(z)=az+b, where a,b∈C and |a|<1; colour map [0,K]-color map with K+1 colors.

Output: Julia set for areaA.

 **i.** $𝐑=𝐦𝐚𝐱\left\{\left|r\right|,\left|b\right|,{\left(2+\left|u\right|\right)}^{\frac{1}{n-1}},{\left(\frac{2+\alpha +\left(1-\alpha \right)\left|u\right|}{1-\alpha }\right)}^{\frac{1}{n-1}}\right\},$

 **ii.** for $z_{0}\in A$ do

 **iii.** j=0

 **iv.** $𝐰𝐡𝐢𝐥𝐞\;\left|z_{j}\right|<R\;$**iv.**and j<K do

 **v.** $y_{j}=\alpha g\left(z_{j}\right)+\left(1-\alpha \right)W\left(z_{j}\right),$

 **vi.** $z_{j+1}=W\left(y_{j}\right),$

 **vii.** j=j+1

 **viii.** $𝐜𝐨𝐥𝐨𝐮𝐫𝐬\;{𝐳}_{0}\;𝐰𝐢𝐭𝐡\;𝐜𝐨𝐥𝐨𝐮𝐫\;𝐦𝐚𝐩\;[j]$

Algorithm 2. Julia set generation for Viscosity approximation process with m-convexity

Input: $W\left(z\right)=z^{n}+uz+r$ where n≥2 𝐚𝐧𝐝 u,r∈C. A ⊂ ℂ-area; K-the maximum number of iterations; α∈(0,1)-parameters for the viscosity approximation iterative with m-convexity where m∈[0, 1]; g(z)=az+b, where a,b∈C and |a|<1; colour map [0,K]-color map with K+1 colors.

Output: Julia set for areaA.

 **i.** $𝐑=𝐦𝐚𝐱\left\{\left|r\right|,\left|b\right|,{\left(2+\left|u\right|\right)}^{\frac{1}{n-1}},{\left(\frac{1+2\alpha }{\left|m\right|\left(1-\alpha \right)}+\left|u\right|+1\right)}^{\frac{1}{n-1}}\right\},$

 **ii.** for $z_{0}\in A$ do

 **iii.** j=0

 **vi.** $𝐰𝐡𝐢𝐥𝐞\;\left|z_{j}\right|<R\;$**iv.**and j<K do

 **v.** $y_{j}=\alpha g\left(z_{j}\right)+m\left(1-\alpha \right)W\left(z_{j}\right),$

 **vi.** $z_{j+1}=W\left(y_{j}\right),$

 **vii.** j=j+1

 **viii.** $𝐜𝐨𝐥𝐨𝐮𝐫𝐬\;{𝐳}_{0}\;𝐰𝐢𝐭𝐡\;𝐜𝐨𝐥𝐨𝐮𝐫\;𝐦𝐚𝐩\;[j]$

Algorithm 3. Julia set generation for Viscosity approximation process with s-convexity

Input: $W\left(z\right)=z^{n}+uz+r$ where n≥2 𝐚𝐧𝐝 u,r∈C. A ⊂ ℂ-area; K-the maximum number of iterations; α∈(0,1)-parameters for the viscosity approximation iterative with s-convexity where s∈(0, 1]; g(z)=az+b, where a,b∈C and |a|<1; colour map [0,K]-color map with K+1 colors.

Output: Julia set for areaA.

 **i.** $𝐑=𝐦𝐚𝐱\left\{\left|r\right|,\left|b\right|,{\left(2+\left|u\right|\right)}^{\frac{1}{n-1}},{\left(\frac{2+s\alpha }{\left(1-s\alpha \right)}+\left|u\right|\right)}^{\frac{1}{n-1}}\right\},$

 **ii.** for $z_{0}\in A$ do

 **iii.** j=0

 **iv.** $𝐰𝐡𝐢𝐥𝐞\;\left|z_{j}\right|<R\;$**iv.**and j<K do

 **v.** $y_{j}=\alpha^{s}g\left(z_{j}\right)+{\left(1-\alpha \right)}^{s}W\left(z_{j}\right),$

 **vi.** $z_{j+1}=W\left(y_{j}\right),$

 **vii.** j=j+1

 **viii.** $𝐜𝐨𝐥𝐨𝐮𝐫𝐬\;{𝐳}_{0}\;𝐰𝐢𝐭𝐡\;𝐜𝐨𝐥𝐨𝐮𝐫\;𝐦𝐚𝐩\;[j]$

### 4.1. Visualizations of Julia set Fractals

**Fig 1:**
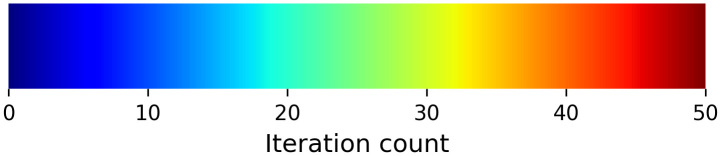
Jet color bar.

In this section, we generate the Julia set fractals by using the viscosity approximation method, viscosity approximation process with m-convexity, and viscosity approximation process with s-convexity. We see the structural behavior of Julia set fractals of these methods. Fig 1, shows the jet colormap.

**Fig 2:**
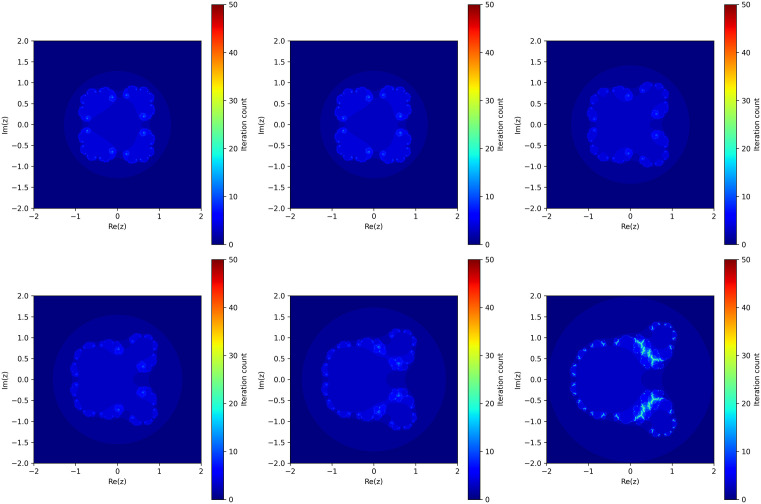
Depict the Julia set fractals with viscosity approximation method by variation of the parameter α.

In Fig 2, the constant parameters are n=4, u=0.1, a=0.9, b=0.5, and r=0.5, while the *α* is varies. Small *α* give rise to compact sets which are highly symmetric and have smooth well-defined boundaries. With further increase of *α* to moderate values, there is a small amount of symmetry breaking and boundary irregularities that can be seen as a gradual growth of nonlinear effects. In larger alpha values, the fractal structures are highly distorted and asymmetric with fragmented regions and roughness of the boundaries, in which they are highly sensitive to initial conditions.

**Fig 3:**
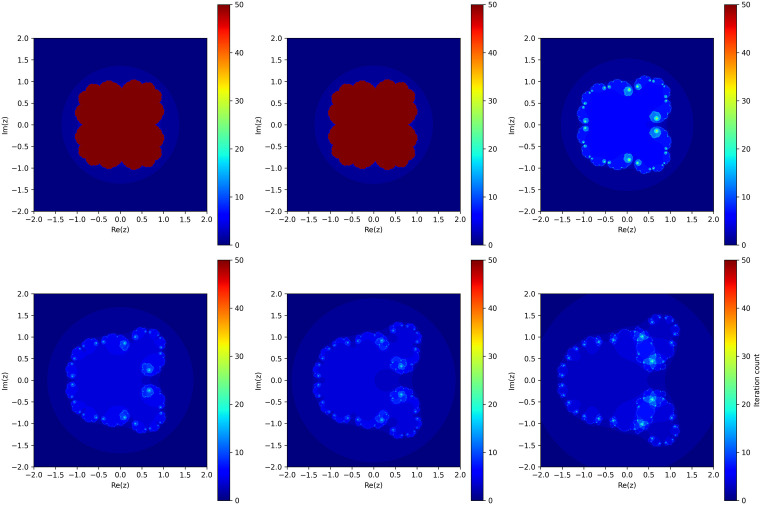
Depict the Julia set fractals with viscosity approximation process with m-convexity by variation of the parameter *α.* In which the value of *m* is 0.7.

Fractals of Julia sets are created through viscosity approximation process with m-convexity are demonstrated in Fig 3. The process applies m-convexity to regulate the process of iteration and affect the fractal behavior. The final fractal structure is dependent on the variation of the parameter α. The values of α are varied whereby more complex boundaries are formed as the value of α increases.

**Fig 4:**
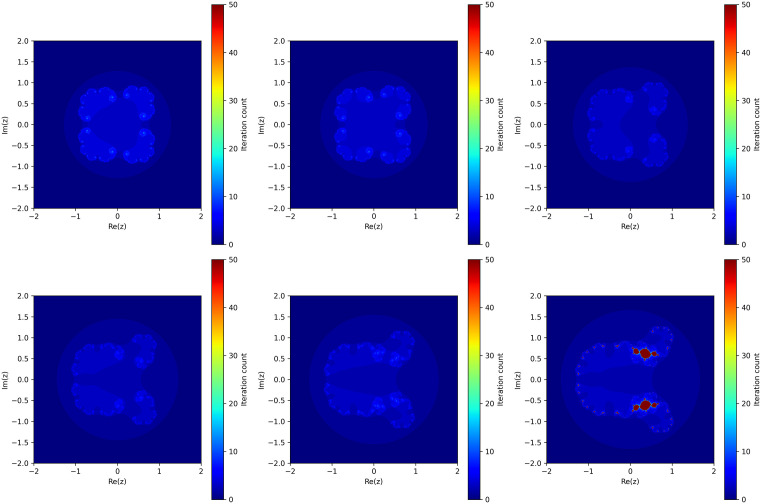
depict the Julia set fractals by using the viscosity approximation method with s-convexity. In which the values of *s* is 0.7.

Figs 2–4 indicate Julia set fractal where it is created by different approximation techniques of viscosity and with different convexity. The basic viscosity approximation method in Fig 2 leads to a less convoluted, less complicated fractal structure with smoother boundaries. Fig 3 presents m-convexity, which enhances the iteration procedure to create a more intricate and defined fractal boundary, which provides a higher level of control on its creation. In Fig 4, the s-convexity is used that further refines the shape of the fractal giving more finer and accurate boundaries than that of Fig 2 and Fig 3. The values of the parameter α are 0.001, 0.01, 0.2, 0.35, 0.5 and 0.65 and the other parameters are constant that are shown in the Table 1.

**Table 1:** variation of the parameter α.

*n*	*u*	*a*	*b*	*r*	*α*
4	0.1	0.9	0.5	0.5	0.001
4	0.1	0.9	0.5	0.5	0.01
4	0.1	0.9	0.5	0.5	0.2
4	0.1	0.9	0.5	0.5	0.35
4	0.1	0.9	0.5	0.5	0.5
4	0.1	0.9	0.5	0.5	0.65

**Fig 5:**
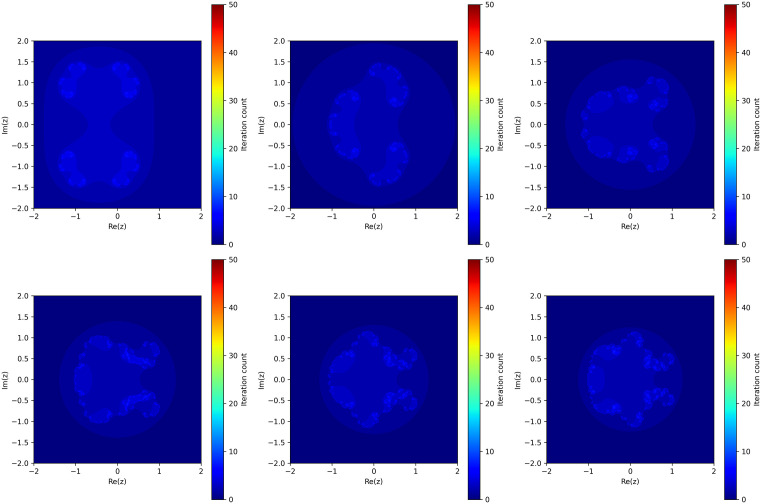
Depict the Julia set fractals by using viscosity approximation method with variation of the parameter *n.*

The Julia set fractal generated with the viscosity approximation method is illustrated in Fig 5. The figure demonstrates the effect different values of *n*. This parameter effict on the structure of Julia set and creates specific visual patterns. The boundary of the fractal changes with the variation of the parameter, as does its degree of complexity and detail.

**Fig 6:**
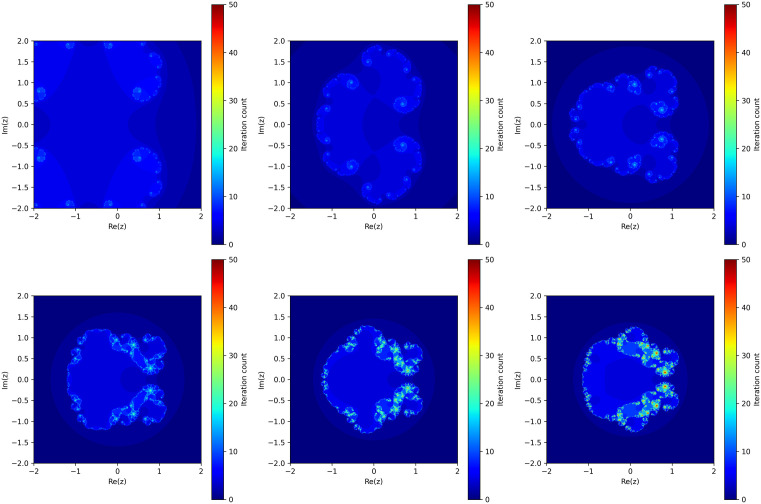
Depict the Julia set fractal by using viscosity approximation process with m-convexity in which the value of *m* is 0.45.

The Julia set fractals in Fig 6, were obtained through a m-convexity process of viscosity approximation. This value illustrates that the addition of m-convexity to the iterative procedure cleanses up the fractal structure to produce finer and detailed boundaries relative to primitive viscosity approximation. It is demonstrated that the change of a given parameter will affect how the fractal looks where large values will add more complexity to the pattern.

**Fig 7:**
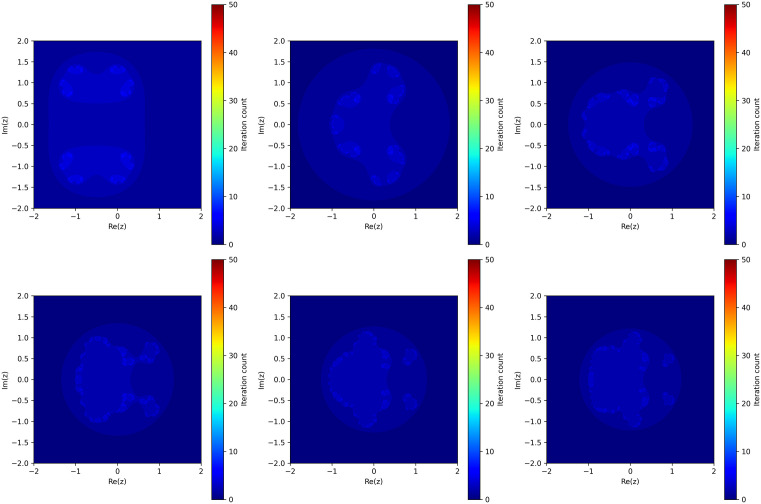
Depict the Julia set fractals by using viscosity approximation method with s-convexity. The value of s is 0.7.

Figs 5–7 are represents Julia set fractals calculated by the various methods of approximation of viscosity. These are shown the Julia set fractal behavior with different values of the parameter *n* while the other parameters *α, u, a, b,* and *r* are constant. The values of the parameter *n* are shown in the Table 2. With the simple calculation of viscosity, it gives a less jagged fractal in Fig 5 with less complex edges. Fig 6, which makes use of the approximation of the viscosity based on m-convexity, gives a more detailed and sharper boundary and gives a better control of the structure of the fractal. The most refined and precise fractal with the sharpest, most correct boundaries is produced by Fig 7 which uses the approximation of viscosity with s-convexity.

**Table 2:** variation of the parameter n.

*n*	*u*	a	*b*	*r*	*α*
2	0.5	0.9	0.5	0.5	0.3
3	0.5	0.9	0.5	0.5	0.3
4	0.5	0.9	0.5	0.5	0.3
5	0.5	0.9	0.5	0.5	0.3
6	0.5	0.9	0.5	0.5	0.3
7	0.5	0.9	0.5	0.5	0.3

**Fig 8:**
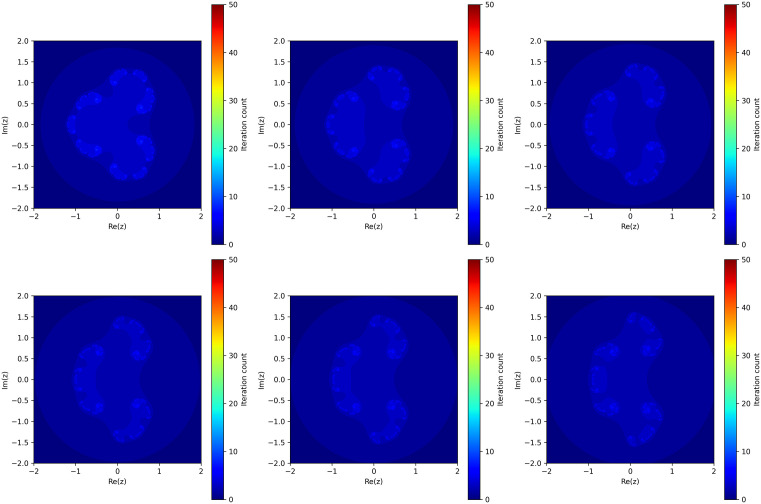
Depict the Jullia set fractals by using the viscosity approximation method with variation of the parameter *u.*

Fig 8 illustrates Julia set fractals produced using the different values of the parameter *u* in six subsections. With the increase in the value of *u* the fractal becomes less smooth, symmetrical. The limits grow more jagged and distorted, more and more complicated and sensitive to the parameter.

**Fig 9:**
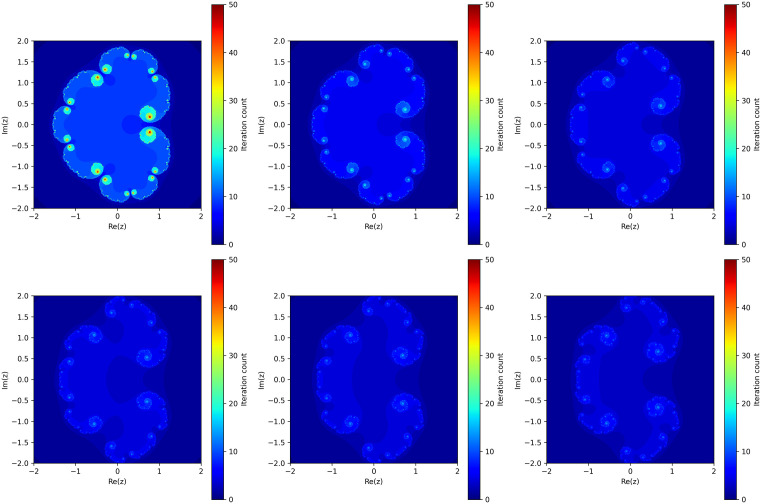
Depict the Julia set fractals by using the viscosity approximation process with m-convexity. In which the value of *m* is 0.4.

Fig 9 shows Julia set fractals created with the viscosity approximation technique of m-convexity and *u* is the parameter that is changed. The larger the *u* value, the more complex is the fractal structure and its boundaries with more defined and sharp edges. The m-convexity allows to improve the procedure of iteration and to better control and refine the structure of the fractal and obtain clearer and more accurate boundaries with increasing parameter.

**Fig 10:**
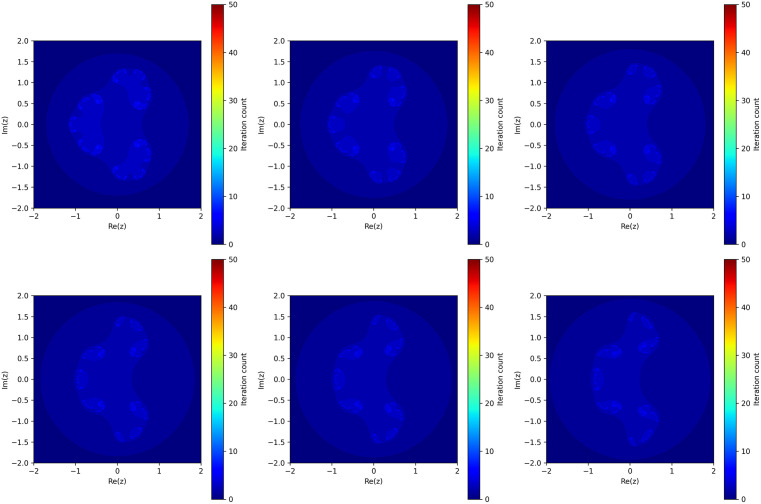
Depict the Julia set fractals by using the viscosity approximation process with s-convexity.

In above figures (Fig 8–9 and Fig 10) the values of *u* are varies from 0.1 to 0.9. The behavior of Julia set fractals with variation of *u* is shows and other parameters are constant. The values of *u* are 0.1, 0.3, 0.45, 0.6, 0.7, and 0.9 are shown in Table 3.

**Table 3:** variation of the parameter u.

*n*	*u*	*a*	*b*	*r*	*α*
3	0.1	0.9	0.5	0.5	0.3
3	0.3	0.9	0.5	0.5	0.3
3	0.45	0.9	0.5	0.5	0.3
3	0.6	0.9	0.5	0.5	0.3
3	0.7	0.9	0.5	0.5	0.3
3	0.9	0.9	0.5	0.5	0.3

**Fig 11:**
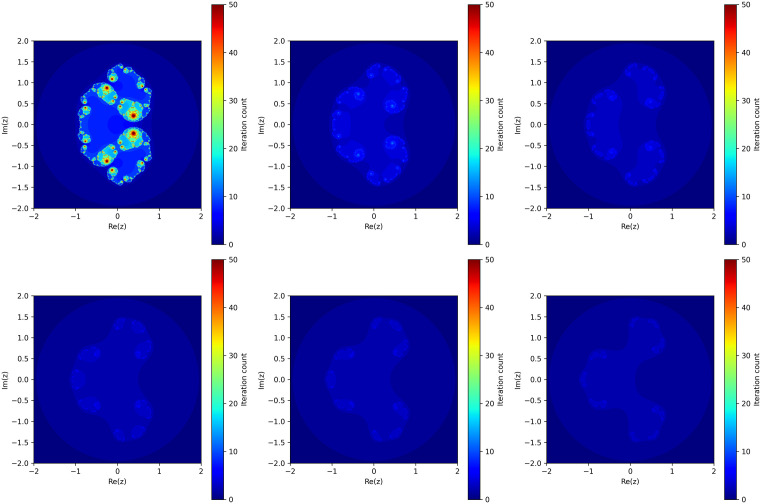
Depict the Julia set fractals by using the viscosity approximation method with variation of the parameter *r.*

Fig 11, shows Julia set fractals obtained through the viscosity approximation technique and different values of the parameter *r*. As *r* increases, the fractal structure also changes. The fractal becomes more complicated where the boundaries are jagged and fragmented.

**Fig 12:**
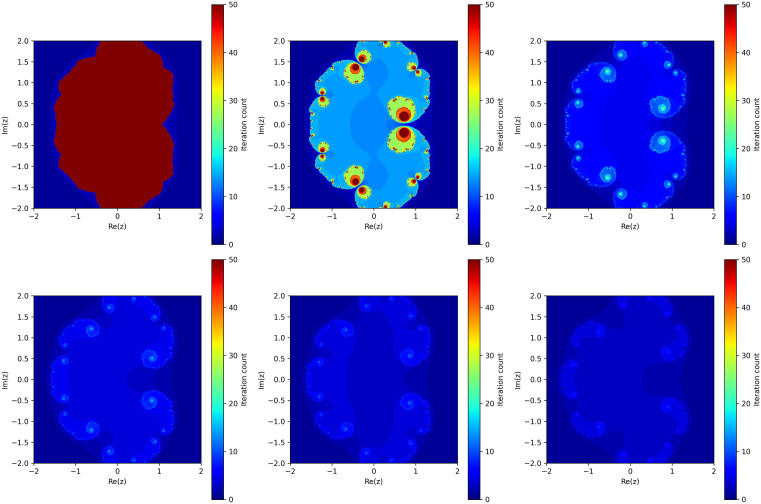
Depict the Julia set fractals by using viscosity approximation process with m-convexity. In which the value of the parameter *m* is 0.3.

Fig 12, shows the behavior of the Julia set fractals with the variation of *r*. The boundaries of the fractal are increasingly disordered and irregular as *r* increases, and are no longer smooth and symmetrical but jagged and fractured. The m-convexity control results in sharped and better-defined boundaries of the fractal.

In above figures, the parameter *r* is varies and other parameters *n, u, a, b* and *α* are constant. The values are shown in Table 4.

**Table 4:** variation of the parameter r.

*n*	*u*	*a*	*b*	*r*	*α*
3	0.5	0.9	0.5	0.1	0.3
3	0.5	0.9	0.5	0.3	0.3
3	0.5	0.9	0.5	0.45	0.3
3	0.5	0.9	0.5	0.6	0.3
3	0.5	0.9	0.5	0.7	0.3
3	0.5	0.9	0.5	0.9	0.3

**Fig 13:**
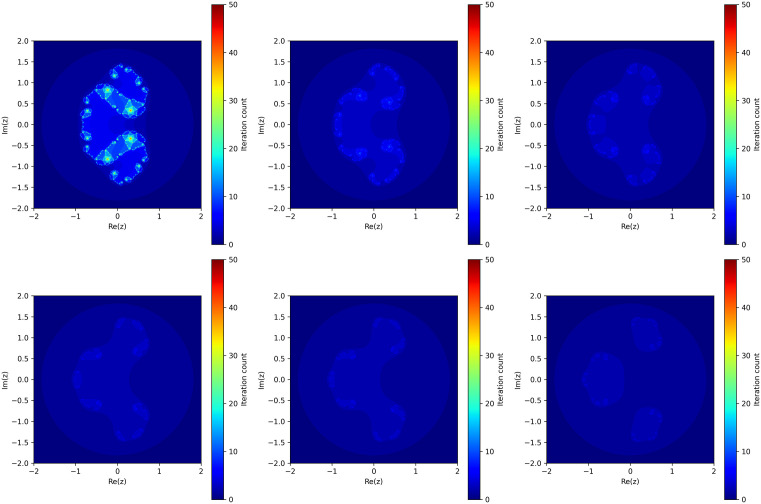
Depict the Julia set fractals by using the viscosity approximation process with s-convexity. The value of *s* is 0.7.

The three figures (Fig 11–12, and Fig 13) all represent Julia set fractals produced with each of the different methods of approximation of the viscosity and each with a different degree of refinement. Fig 11 is the fractal generated using the basic method of the viscosity approximation. Fig 12 uses the m-convexity method, which offers sharper and more defined boundaries and the fractal is more complex as the *r* parameter is larger. This approach is more controlling to the shape of the fractal in relation to the simple approach in Fig 11. Fractal further refinements the fractal is further refined in Fig 13 using s-convexity giving the most detailed and sharply defined boundaries.

## 5. Algorithm for Mandelbrot set Fractals

In this section, we discuss the algorithms for the Mandelbrot set fractals by using the viscosity approximation method, viscosity approximation process with m-convexity, and viscosity approximation process with s-convexity.

**Algorithm 4. For Viscosity Approximation process**

Input: $W(z)=z^{n}+uz+r$ **where** n≥2 and u,r∈C. **A ⊂ ℂ-area; K-the maximum number of iterations;** α∈(0,1)**-parameters for the viscosity approximation iterative method;** g(z)=az+b, **where** a,b∈C **and** |a|<1; **colour map [0,K]-color map with** K+1 **colors.**

**Output: Mandelbrot set for area** A.

 **i.** $\text{R}=\text{max}\left\{|r|,|b|,\;{\left(\text{2}+|u|\right)}^{\frac{\text{1}}{n-\text{1}}},\;{\left(\frac{\text{2}+\alpha +(\text{1}-\alpha )|u|}{\text{1}-\alpha }\right)}^{\frac{\text{1}}{n-\text{1}}}\right\},$

 **ii. for** r∈A **do**

 **iii.** j=0

 **iv.** $z_{\text{0}}=r$

 **v.** $\text{while }\left|z_{j}\right|<R\;$**v.and** j<K do

 **vi.** $y_{j}=\alpha g\left(z_{j}\right)+(\text{1}-\alpha )W\left(z_{j}\right),$

 **vii.** $z_{j+\text{1}}=W\left(y_{j}\right),$

 **viii.** j=j+1

**Algorithm 5. For Viscosity Approximation process with m-convexity**

Input: $W(z)=z^{n}+uz+r$ **where** n≥2 and u,r∈C. **A ⊂ ℂ-area; K-the maximum number of iterations;** α∈(0,1)**-parameters for the viscosity approximation iterative process with m-convexity where** m∈[0, 1]; g(z)=az+b, **where** a,b∈C **and** |a|<1; **colour map [0,K]-color map with** K+1 **colors.**

**Output: Mandelbrot set for area** A.

 **i.** $R_{\text{1}}={\left(\frac{\text{1}+\text{2}\alpha }{\left|m|(\text{1}-\alpha \right)}+|u|+\text{1}\right)}^{\frac{\text{1}}{n-\text{1}}},$

 **ii.** $\text{R}=\text{max}\left\{|r|,|b|,\;{\left(\text{2}+|u|\right)}^{\frac{\text{1}}{n-\text{1}}},\;R_{\text{1}}\right\},$

 **iii. for** r∈A **do**

 **iv.** j=0

 **v.** $z_{\text{0}}=r$

 **vi.** $\text{while }\left|z_{j}\right|<R\;$ **and**  j<K do

 **vii.** $y_{j}=\alpha g\left(z_{j}\right)+m(\text{1}-\alpha )W\left(z_{j}\right),$

 **viii.** $z_{j+\text{1}}=W\left(y_{j}\right),$

 **ix.** j=j+1

 **x.** $\text{colours }{\text{z}}_{\text{0}}\text{ with colour map }[j]$

**Algorithm 6. For Viscosity Approximation process with s-convexity**

Input: $W(z)=z^{n}+uz+r$ where n≥2 and u,r∈C. A ⊂ ℂ-area; K-the maximum number of iterations; α∈(0,1)-parameters for the viscosity approximation iterative process with s-convexity where s∈(0, 1]; g(z)=az+b, where a,b∈C and |a|<1; colour map [0,K]-color map with K+1 colors.

Output: Mandelbrot set for areaA.

 **i.** $R_{\text{1}}={\left(\frac{\text{2}+s\alpha }{\left(\text{1}-s\alpha \right)}+|u|\right)}^{\frac{\text{1}}{n-\text{1}}},$

 **ii.**$\text{R}=\text{max}\left\{|r|,|b|,\;{\left(\text{2}+|u|\right)}^{\frac{\text{1}}{n-\text{1}}},\;R_{\text{1}}\right\},$

 **iii. for** r∈A **do**

 **iv.** j=0

 **v.** $z_{\text{0}}=r$

 **vi.** $\text{while }\left|z_{j}\right|<R\;$ **and** j<K do

 **vii.** $y_{j}=\alpha^{s}g\left(z_{j}\right)+(\text{1}-\alpha {)}^{s}W\left(z_{j}\right),$

 **viii.** $z_{j+\text{1}}=W\left(y_{j}\right),$

 **ix.** j=j+1

 **x.** $\text{colours }{\text{z}}_{\text{0}}\text{ with colour map }[j]$

### 5.1. Visualizations of Mandelbrot set fractals

In this, we generate the Mandelbrot set fractals via viscosity approximation method, viscosity approximation process with m-convexity, and viscosity approximation process with s-convexity. We see the structural behavior of Mandelbrot set fractals of these methods.

**Fig 14:**
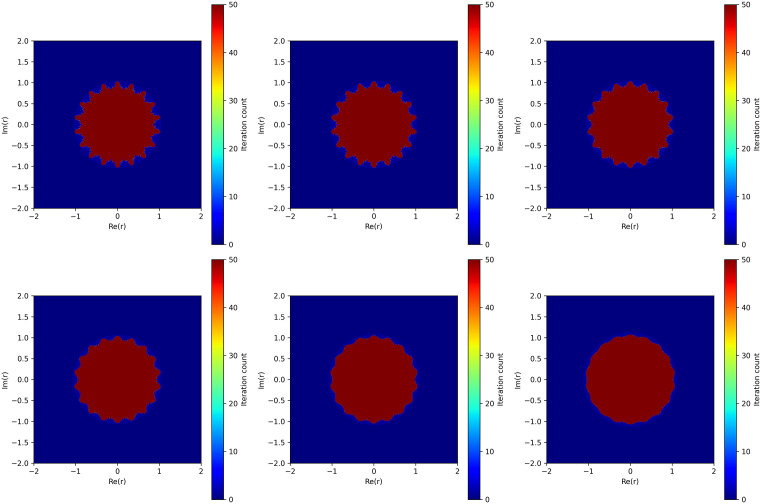
Depict Mandelbrot set fractals using viscosity approximation method for different values of *α.*

In Fig 14, the Mandelbrot set fractal has less complexity and smoother and more symmetrical boundaries at lower values of *α*. The regularities are clear and foreseeable; besides, the fractal structure is not very complicated. When *α* rises to moderate levels, the fractal symmetry starts to ruin leading to a few boundary anomalies and an intricate structure.

**Fig 15:**
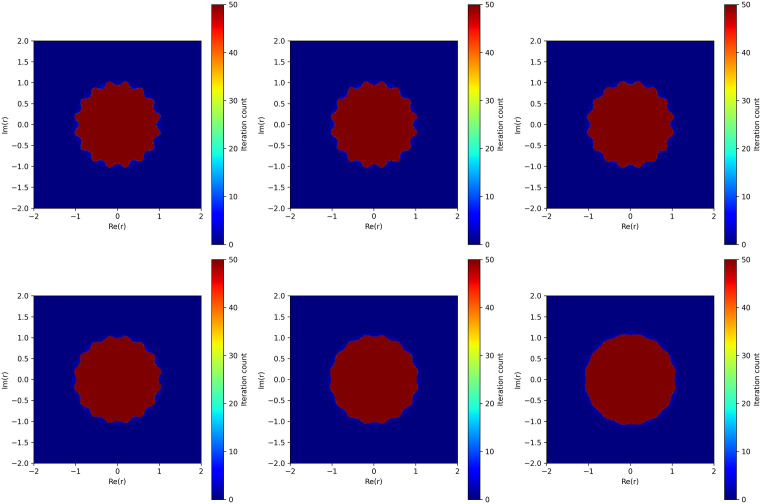
Depict the Mandelbrot set fractals by using the viscosity approximation process with m-convexity. The values of *m* is 0.75.

In Fig 15, Mandelbrot set fractals obtained by the viscosity approximation method when using *m*-convexity, the parameter of *α* plays a significant role in determining the structure of the fractal. The fractal is more symmetrical and smoother at smaller values of *α,* and otherwise resembles fractals produced with the viscosity approximation technique, but with finer detail because of the extra *m*-convexity. The higher the value of *α,* the more fractal boundaries are irregular with sharp and more defined edges. The existence of m-convexity enables more control of the iteration process resulting in fractals with more detailed and complex boundary structures in comparison to the smoother ones at lower alpha values. The fractals become even more complex at larger values of *α* where the boundaries become highly jagged and fragmented and asymmetric. The dark regions indicate magnitude divergence or stabilization. The dark regions represent where the magnitude of the function diverges (escape regions) or converges to a fixed value.

**Fig 16:**
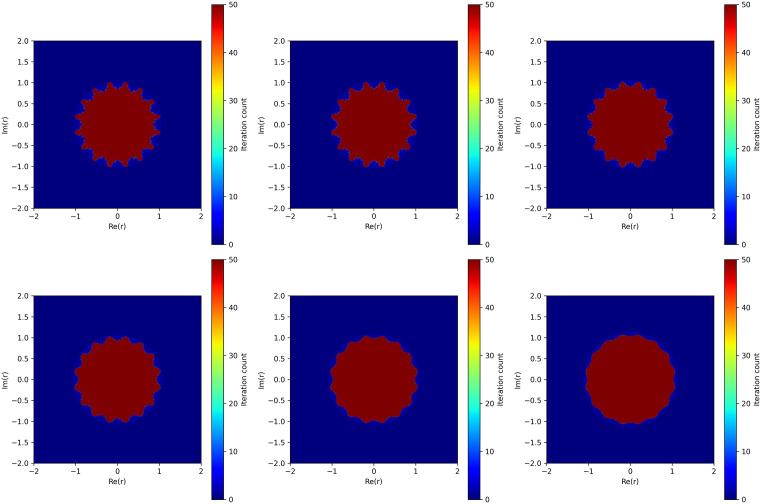
Depict the Mandelbrot set fractals by using the viscosity approximation process with s-convexity. The value of *s* is 0.99.

Figs 14–16 show Mandelbrot set fractals obtained by the viscosity approximation method with the varying degrees of refinement, namely the change in the parameter *α.* Fig 14, illustrates the fractals formed with basic viscosity approximation technique in which variations in *α* cause the formation of smoother and less complicated boundaries and the fractal structure is relatively simple and symmetrical. Fig 15 uses the approximation of viscosity technique that uses m-convexity, where the larger *α* the sharper and more defined the boundaries become, and thus the fractal becomes more complex and not so symmetrical as in Fig 14. Fig 16 employs the viscosity approximation technique that is based on s-convexity. The fractal structures increase in complexity with the value of *α* in these figures with both techniques of m-convexity and s-convexity contributing extra levels of control and detail to the Mandelbrot set fractals. The parameter *α* is varies from 0.05 to 0.7 and the other parameters are fixed. The values of the parameter *α* are shown in Table 5.

**Table 5:** variation of the parameter α.

*n*	*u*	*a*	*b*	*α*
17	0.001	0.08	0.001	0.05
17	0.001	0.08	0.001	0.1
17	0.001	0.08	0.001	0.2
17	0.001	0.08	0.001	0.3
17	0.001	0.08	0.001	0.5
17	0.001	0.08	0.001	0.7

**Fig 17:**
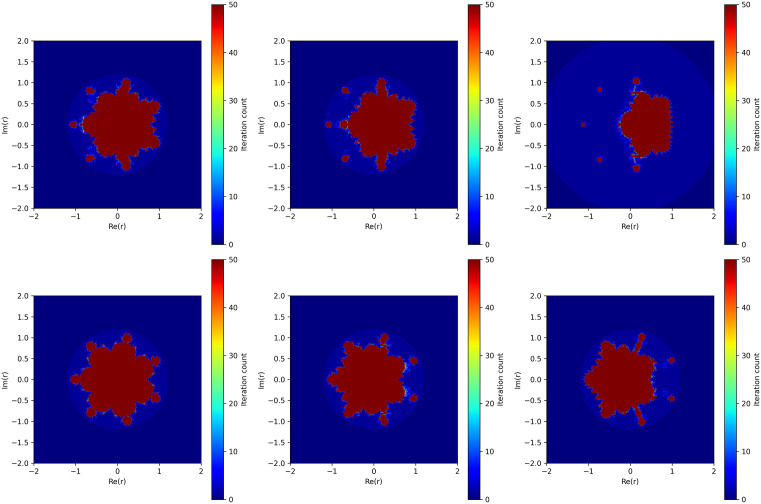
Depict Mandelbrot set fractals by using the viscosity approximation method for different values of *b.*

Fig 17, shows the behavior of Mandelbrot set fractals by variation of the parameter b. At smaller values of b, the fractals tend to have more rounded and smoother edges, more simple structures and less complexity. These fractals are more predictable and regular and have a distinct shape and least distortion. The lines of the fractal begin to take on irregularities as *b* increases. The fractals are changing to become jagged and craggy and more complex patterns are formed.

**Fig 18:**
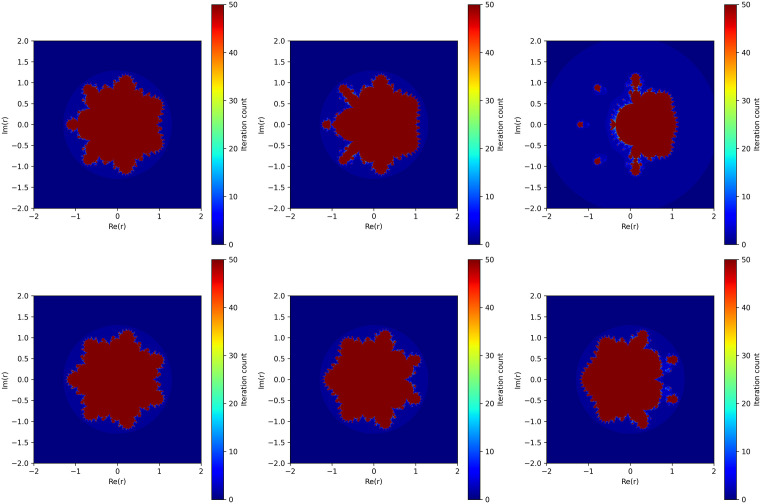
Depict the Mandelbrot set fractals by using the viscosity approximation method with m-convexity. The value of *m* is 0.5.

Fig 18 produces fractals of Mandelbrot set by applying viscosity approximation method with m-convexity as the parameter *b* is varied. The fractals drastically change in structure due to *b* change. At smaller values of *b*, the fractals are relatively smooth having defined and symmetrical edges. The degree of complexity is minimal as well as the whole structure is simple and presents fewer distortions and fewer fragments. The fractals start having more irregularities, and more defined edges, as the *b* increases.

**Fig 19:**
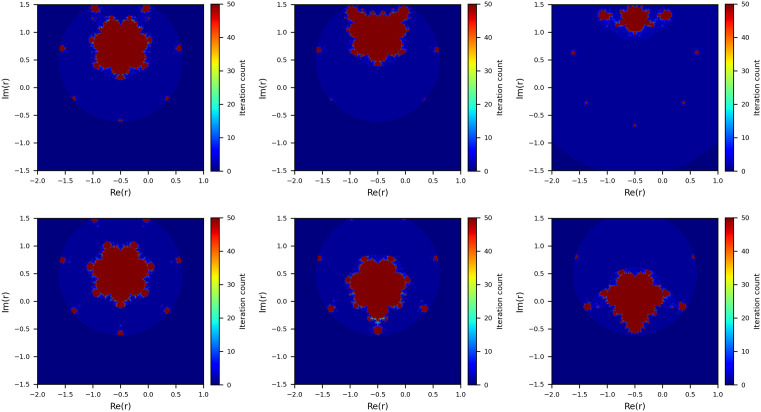
Depict the Mandelbrot set fractals via viscosity approximation process with s-convexity. The values of *s* is 0.2.

Above Figs 17–19, shows the behavior of Mandelbrot set fractals with variation of the parameter *b* while other parameters *n, u, a, α* are constant. Fig 17 includes smoother and simpler boundaries of the fractals, where *b* results in minor irregularities yet a fairly uniform structure. with the m-convexity method, the boundaries become sharper and more defined with *b*: Fig 18 comes up with more complex and detailed fractals. Fig 19 once again applies the s-convexity method, resulting in the most complex and broken edges, with the greatest degree of accuracy and control. With increasing *b*, the fractals tend to take simpler forms in Fig 17 to complex and intricate forms in Figs 18 and 19. The values of the parameter b are −0.5, −1, −2.1, 0.001, 0.5 and 1 which are shown in Table 6.

**Table 6:** variation of the parameter b.

*n*	*u*	*a*	*b*	*α*
8	0.001	0.99	−0.5	0.35
8	0.001	0.99	−1	0.35
8	0.001	0.99	−2.1	0.35
8	0.001	0.99	0.001	0.35
8	0.001	0.99	0.5	0.35
8	0.001	0.99	1	0.35

**Fig 20:**
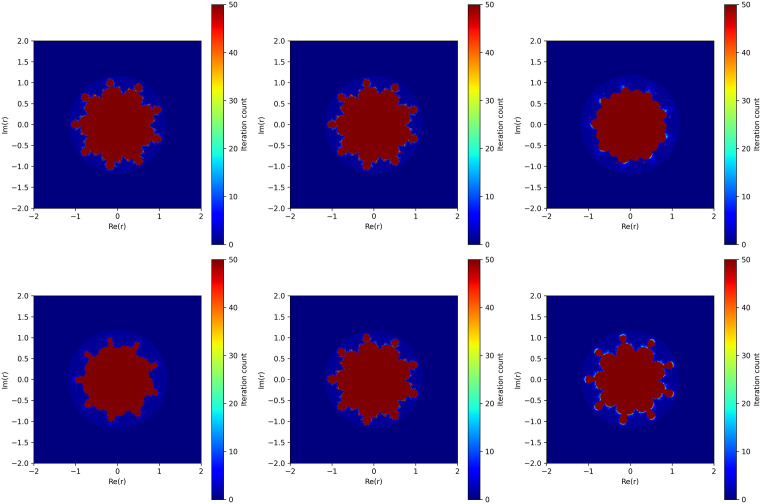
Depict the Mandelbrot set fractals using the viscosity approximation method with different values of *u.*

In Fig 20, the change in the value of the parameter *u* in Mandelbrot set fractals greatly influences the way within which they look and their complexity. Varying the parameter *u*, the fractals are transformed in their structure in terms of boundary, complexity and symmetry. The fractals have a smoother and more symmetrical edges at lower value of the parameter *u*. When *u* is increase, the fractals start to have more inconsistencies at their edges. The boundaries are more jagged and torn and the fractal structure is more complex and detailed.

**Fig 21:**
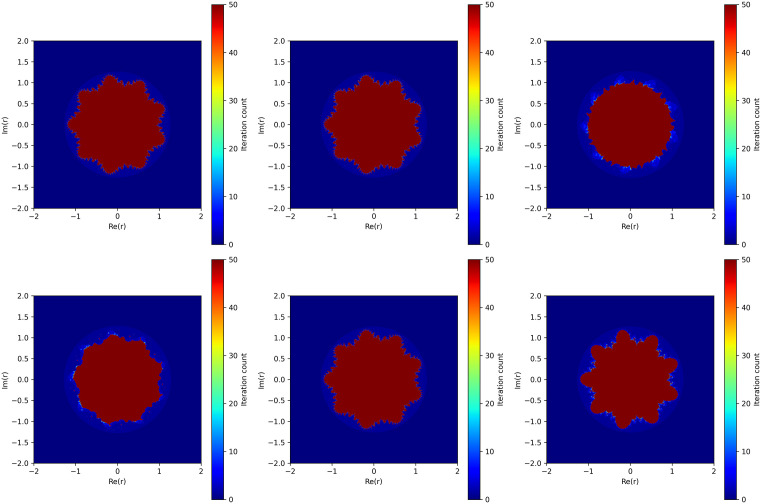
Depict the Mandelbrot set fractals by using the viscosity approximation process with m-convexity. The value of the parameter *m* is 0.4.

Fig 21, illustrates how the change of *u* causes Mandelbrot set fractal to take smooth symmetrical shapes with lower values and jagged, irregular and chaotic shapes with high values. As *u* increases, the fractals get more intricate and sensitive to initial conditions with more details in the boundary and more intricate in general.

**Fig 22:**
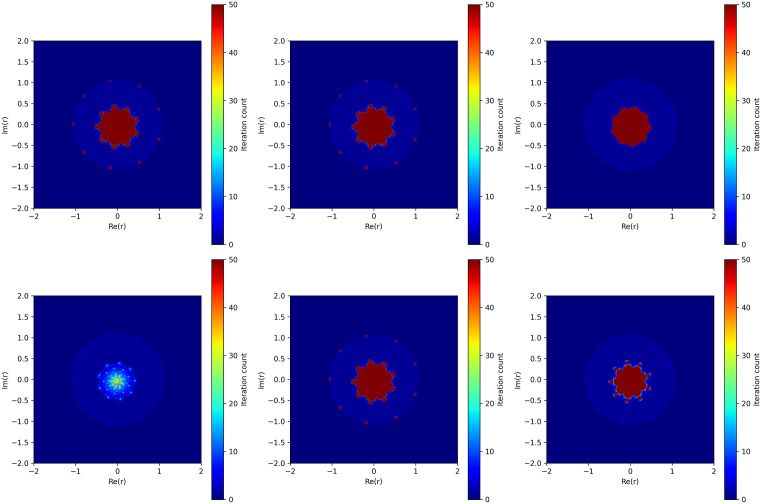
Depict the Mandelbrot set fractals by using the viscosity approximation method with s-convexity. The value of *s* is 0.001.

Above figures (Fig 20–21, and Fig 22), shows the behavior of Mandelbrot set fractals with variation of the parameter *u* while other parameters are fixed. The values are shown in Table 7.

**Table 7:** variation of the parameter u.

*n*	*u*	*a*	*b*	*α*
10	0.001i	0.9	0.1i	0.4
10	−0.005i	0.9	0.1i	0.4
10	−0.6i	0.9	0.1i	0.4
10	0.9i	0.9	0.1i	0.4
10	0.00005i	0.9	0.1i	0.4
10	0.1	0.9	0.1i	0.4

**Fig 23:**
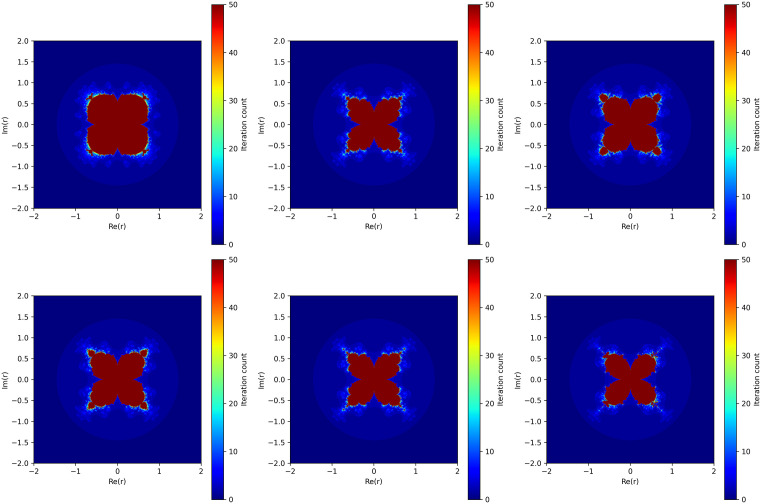
Depict the Mandelbrot set fractals by using the viscosity approximation method with different values of *a.*

Fig 23 depicts the behavior of Mandelbrot set fractals obtained by the viscosity approximation method. In which the parameter *a* varies, while the other values remain constant. The Mandelbrot set fractals become denser as the value of the parameter *a* is negative, and the size of the petal’s changes when the parameter *a* changes. Small values of the parameter *a* cause the petals to spread, whereas big values of the parameter *a* cause the petals to shrink.

**Fig 24:**
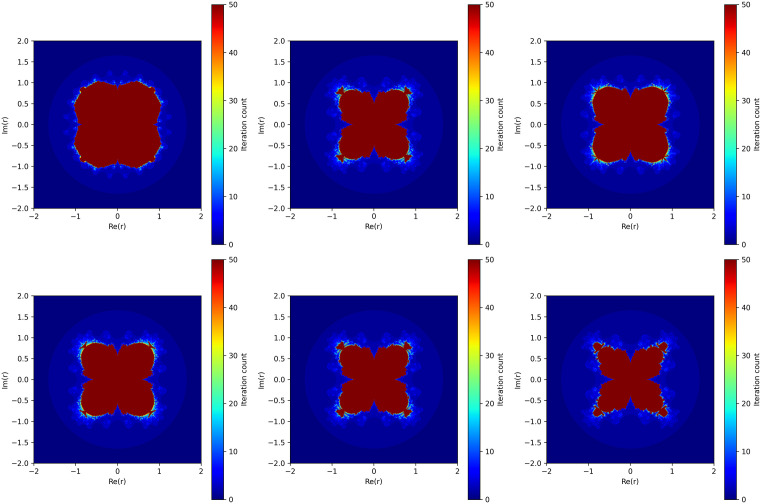
Depict the Mandelbrot set fractals by using viscosity approximation process with m-convexity. In which the value of the parameter *m* is 0.5.

Fig 24, shows the behavior of Mandelbrot set fractals by using the viscosity approximation process with m-convexity. In which the value of the parameter *m.* In which the parameter *a* is varies and the value of the parameter *m* is 0.5 while other parameters are constant. At negative value of the *a* the Mandelbrot set fractals is denser and change the value of the parameter *a* the petals size is change. The small values of the parameter *a* the petals are spread and the large value of the parameter the petals shrink.

**Fig 25:**
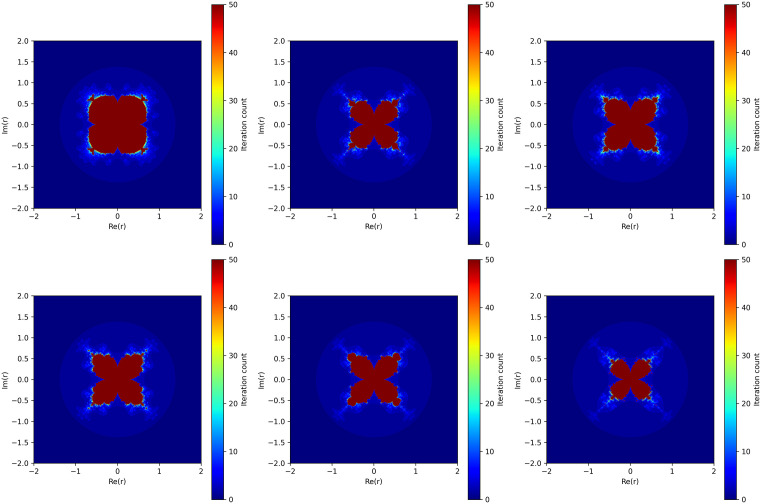
Depict the Mandelbrot set fractals by using the viscosity approximation approach with s-convexity. The value of *s* is 0.7.

In the above figures (Fig 23–24, and Fig 25), the parameter *a* varies across the six subsections −0.9,0.5,0.01,0.2,0.6, and 0.99. The other parameters are n = 5, u = 0.5, b = −0.01 and α = 0.4 are constant. The values are shown in Table 8.

**Table 8:** variation of the parameter *a.*

*n*	*u*	*a*	*b*	*α*
5	0.5	−0.9	−0.01	0.4
5	0.5	0.5	−0.01	0.4
5	0.5	0.01	−0.01	0.4
5	0.5	0.2	−0.01	0.4
5	0.5	0.6	−0.01	0.4
5	0.5	0.99	−0.01	0.4

**Fig 26:**
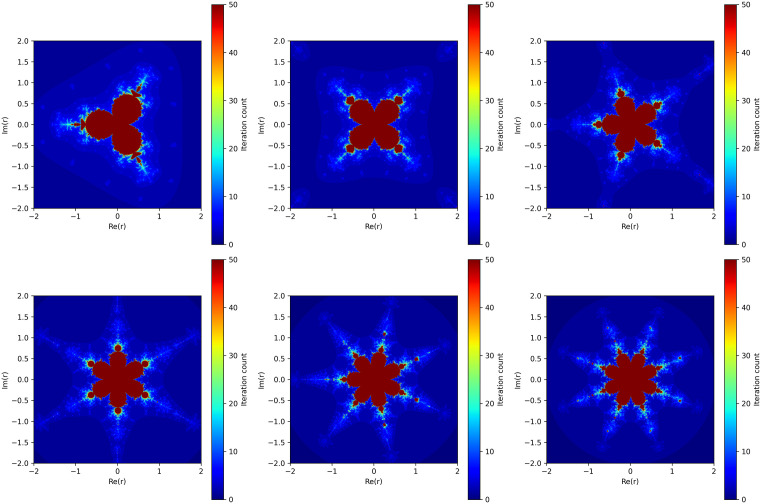
Depict the Mandelbrot set fractals by using viscosity approximation method with different values of the parameter *n.*

In Fig 26, fractal of Mandelbrot set fractals is constructed by the viscosity approximation method with different value of the parameter *n*. The variation in *n* prominently changes the structure of the fractal particularly with reference to the sharpness of boundaries, and complicated structure. The fractals have smoother and more symmetrical edges at lower values of *n*. The edges are smooth and continuous and this adds to a less disordered and more organized view of the fractal. The fractals are very complicated at large values of *n* and their boundaries are extremely irregular, fragmented, and asymmetric. The sharpness of the borders and sensitivity of the fractals to the initial conditions is enhanced, which is why the slightest alterations in initial values cause radically different patterns.

**Fig 27:**
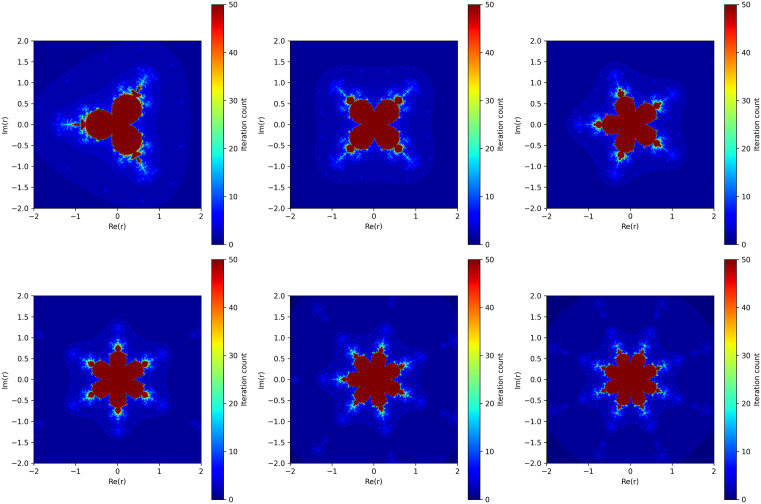
Depict the Mandelbrot set fractals by using viscosity approximation methos with m-convexity. The value of the parameter *m* is 0.4.

In Fig 27, fractal of Mandelbrot set fractals is constructed by the viscosity approximation method with *m*-convexity. In this figure the parameter *n* is varies and the other parameters are fixed. The variation in *n* prominently changes the structure of the fractal particularly with reference to the sharpness of boundaries, and complicated structure. The fractals are very complicated at large values of *n* and their boundaries are extremely irregular, fragmented, and asymmetric. The sharpness of the borders and sensitivity of the fractals to the initial conditions is enhanced, which is why the slightest alterations in initial values cause radically different patterns. When increase the value of the parameter *n* the petals are increase.

**Fig 28:**
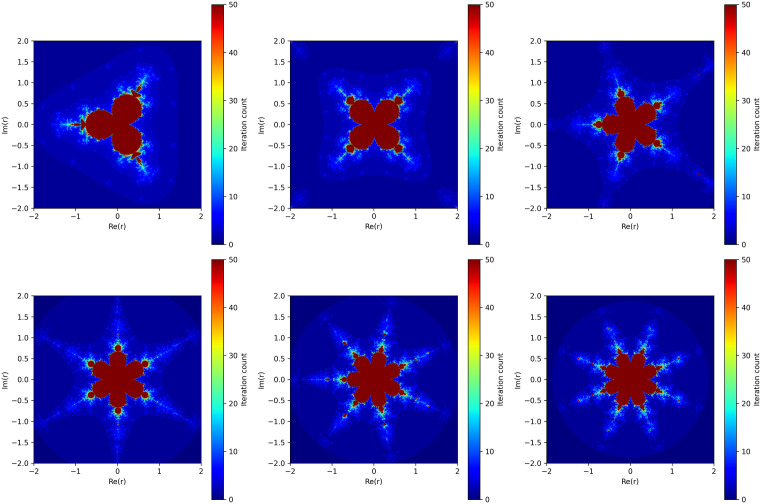
Depict the Mandelbrot set fractals for viscosity approximation process with s-convexity. In which the value of *s* is 0.99.

In the above figures (Fig 26–27, and Fig 28), only variation across the six subplots is the degree of the complex polynomial *n* which varies from 4 to 9 while other parameters are constant. The values of all the parameters are shown in Table 9.

**Table 9:** variation of the parameter n.

*n*	*u*	*a*	*b*	*α*
4	0.5	0.9	−0.01	0.99
5	0.5	0.9	−0.01	0.99
6	0.5	0.9	−0.01	0.99
7	0.5	0.9	−0.01	0.99
8	0.5	0.9	−0.01	0.99
9	0.5	0.9	−0.01	0.99

## 6. Generation of Biomorph

The biomorphs are altered Julia sets that were proposed by Pickover [13] and are commonly known to produce distinct shapes. Pickover discovered the natural laws within which living things exist yet did so more in the realms of mysticism rather than scientifically. In any case, biomorphs are sightseeing and possess unique shapes. Pickover created a collection of biomorphs which resembles single-celled organisms which are believed to be primordial, through numerous and complex techniques and parameters. The forms resemble biological objects, such as microbes and with fine parameter tuning, can also be used to create structures such as cell walls, membranes, nuclei, and organelles. When Julia and Mandelbrot set deal with the mathematical structures and the complicated patterns created by repeated calculations, biomorphs provide a unique insight. They bring in the new creative and evolutionary factor, employing genetic algorithms to emulate the biologic processes of development, mutation, and selection. The effect of this strategy is the creation of shapes that are visually interesting and mirror evolution in nature. Biomorphs combine computational principles with chance in making unpredictable and intriguing results. They broaden the opportunities of both aesthetic and scientific investigation of fractals, and provide an approach to exploring emergent behavior and aesthetic decisions that the more stringent, deterministic nature of more traditional fractals excludes.

### 6.1. Algorithm for Generation of Biomorph

In this section, we discuss the algorithms for biomorph by using the viscosity approximation method, viscosity approximation process with m-convexity, and viscosity approximation process with s-convexity.

Algorithm 7. For Viscosity approximation process

Input: $W(z)=z^{n}+uz+r$ **where** n≥2 and u,r∈C. **A ⊂ ℂ-area; K-the maximum number of iterations;** α∈(0,1)**-parameters for the viscosity approximation iterative method;** g(z)=az+b, **where** a,b∈C **and** |a|<1; **colour map [0,K]-color map with** K+1 **colors.**

Output: **Biomorph for area**A.

 **i.** $\text{R}=\text{max}\left\{|r|,|b|,{\left(\text{2}+|u|\right)}^{\frac{\text{1}}{n-\text{1}}},{\left(\frac{\text{2}+\alpha +(\text{1}-\alpha )|u|}{\text{1}-\alpha }\right)}^{\frac{\text{1}}{n-\text{1}}}\right\},$

 **ii. for** $z_{\text{0}}\in A$ **do**

 **iii.** j=0

 **iv.** $\text{while }\left|z_{j}\right|<R\;$ **and** j<K do

 **v.** $y_{j}=\alpha g\left(z_{j}\right)+(\text{1}-\alpha )W\left(z_{j}\right),$

 **vi.** $z_{j+\text{1}}=W\left(y_{j}\right),$

 **vii.** j=j+1

 **viii. If** |Re(z)|<R **or** |Im(z)|<R, **then**

 **ix.** $\text{colours }{\text{z}}_{\text{0}}\text{ with colour map }[j]$

 **x. else**

 **xi.** $\text{colours }{\text{z}}_{\text{0}}\text{ with colour map }[\text{0}]$

Algorithm 8. For Viscosity approximation process with m-convexity

Input: $W(z)=z^{n}+uz+r$ **where** n≥2 and u,r∈C. **A ⊂ ℂ-area; K-the maximum number of iterations;** α∈(0,1)**-parameters for the viscosity approximation iterative with m-convexity where** m∈[0, 1]; g(z)=az+b, **where** a,b∈C **and** |a|<1; **colour map [0,K]-color map with** K+1 **colors.**

Output: **Biomorph for area**A.

 **ix.** $\text{R}=\text{max}\left\{|r|,|b|,{\left(\text{2}+|u|\right)}^{\frac{\text{1}}{n-\text{1}}},{\left(\frac{\text{1}+\text{2}\alpha }{\left|m|(\text{1}-\alpha \right)}+|u|+\text{1}\right)}^{\frac{\text{1}}{n-\text{1}}}\right\},$

 **x. for** $z_{\text{0}}\in A$ **do**

 **xi.** j=0

 **xii.** $\text{while }\left|z_{j}\right|<R\;$**and** j<K do

 **xiii.** $y_{j}=\alpha g\left(z_{j}\right)+m(\text{1}-\alpha )W\left(z_{j}\right),$

 **xiv.** $z_{j+\text{1}}=W\left(y_{j}\right),$

 **xv.** j=j+1

 **xvi. If** |Re(z)|<R **or** |Im(z)|<R, **then**

 **xvii.** $\text{colours }{\text{z}}_{\text{0}}\text{ with colour map }[j]$

 **xviii. else**

 **xix.** $\text{colours }{\text{z}}_{\text{0}}\text{ with colour map }[\text{0}]$

Algorithm 9. For Viscosity approximation process with s-convexity

Input: $W(z)=z^{n}+uz+r$ **where** n≥2 and u,r∈C. **A** ⊂ **ℂ-area; K-the maximum number of iterations;** α∈(0,1)**-parameters for the viscosity approximation iterative with s-convexity where** s∈(0, 1]; g(z)=az+b, **where** a,b∈C **and** |a|<1; **colour map [0,K]-color map with** K+1 **colors.**

Output: **Biomorph for area**A.

 **ix.** $\text{R}=\text{max}\left\{|r|,|b|,{\left(\text{2}+|u|\right)}^{\frac{\text{1}}{n-\text{1}}},{\left(\frac{\text{2}+s\alpha }{\left(\text{1}-s\alpha \right)}+|u|\right)}^{\frac{\text{1}}{n-\text{1}}}\right\},$

 **x. for** $z_{\text{0}}\in A$ **do**

 **xi.** j=0

 **xii.** $\text{while }\left|z_{j}\right|<R\;$ **and** j<K do

 **xiii.** $y_{j}=\alpha^{s}g\left(z_{j}\right)+(\text{1}-\alpha {)}^{s}W\left(z_{j}\right),$

 **xiv.** $z_{j+\text{1}}=W\left(y_{j}\right),$

 **xv.** j=j+1

 **xvi. If** |Re(z)|<R **or** |Im(z)|<R, **then**

 **xvii.** $\text{colours }{\text{z}}_{\text{0}}\text{ with colour map }[j]$

 **xviii. else**

 **xix.** $\text{colours }{\text{z}}_{\text{0}}\text{ with colour map }[\text{0}]$

### 6.2. Visualizations of Biomorphs

We generate the biomorph by using the viscosity approximation method, viscosity approximation process with m-convexity, and viscosity approximation process with s-convexity.

**Fig 29:**
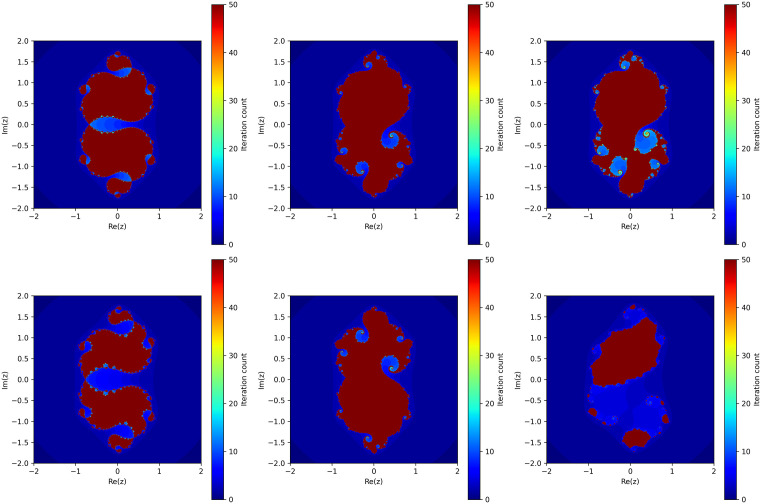
Depict the behavior of Biomorph by using the viscosity approximation method.

Fig 29, shows fractures of Biomorph using the method of viscosity approximation when changing the values of the parameter *b*. The value of *b* varies and it has a significant effect on the structure of the fractal, especially its boundary characteristics, complexity and the overall visual appearance of the fractal. Fractals with smooth and more symmetrical boundaries are observed to occur at lower *b*. As the value of *b* increases, the fractals become very complicated and chaotic.

**Fig 30:**
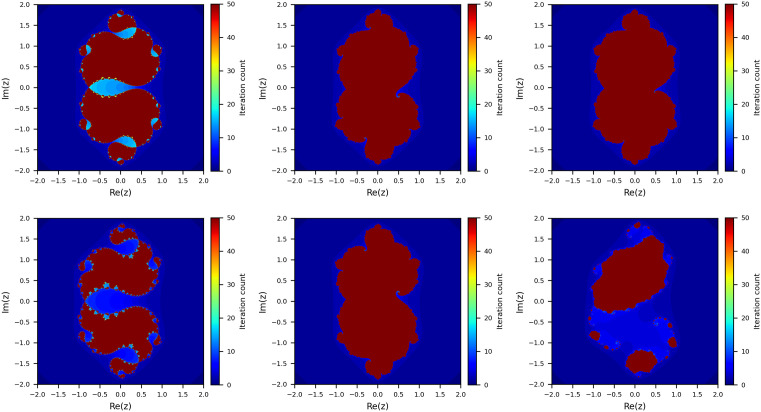
Depict the Biomorph by using the viscosity approximation method with m-convexity. In which the value of *m* is 0.6.

When the values of the parameter *b* are changed, Fig 30 displays fractures of Biomorph utilizing the viscosity approximation approach with m-convexity. The fractal’s structure, particularly its border properties, intricacy, and overall visual appeal, are significantly impacted by the fluctuating value of *b*. It is noted that fractals with more symmetrical and smooth borders appear with lower *b*. The fractals get more complex and chaotic as the value of *b* rises.

**Fig 31:**
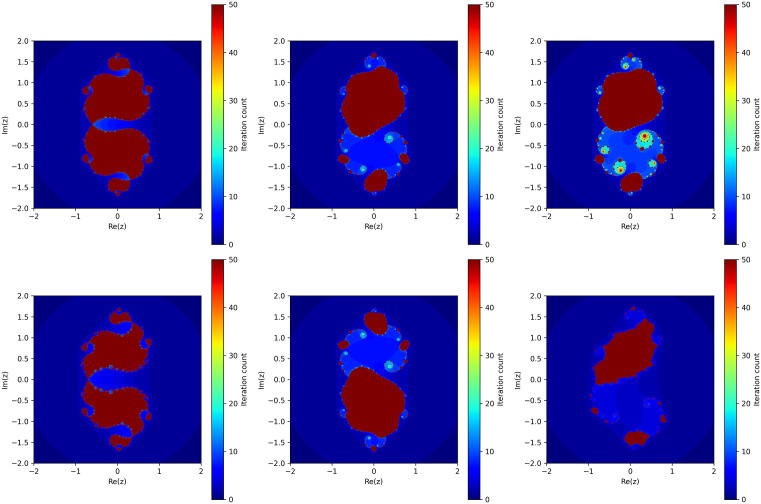
Shows the behavior of Biomorph by using the viscosity approximation method with s-convexity. The value of *s* is 0.82.

In the above figures (Fig 29–30, and Fig 31), only variation in parameter *b* across the six subplots. While the other parameters are constant. The values of all the parameters are shown in Table 10.

**Table 10:** Variation of the parameter b.

*n*	*u*	*a*	*r*	*b*	*α*
3	0.9	0.6	0.1	0.001	0.5
3	0.9	0.6	0.1	0.1i	0.5
3	0.9	0.6	0.1	−0.05 + 0.05i	0.5
3	0.9	0.6	0.1	0.2	0.5
3	0.9	0.6	0.1	−0.1i	0.5
3	0.9	0.6	0.1	0.5 + 0.3i	0.5

**Fig 32:**
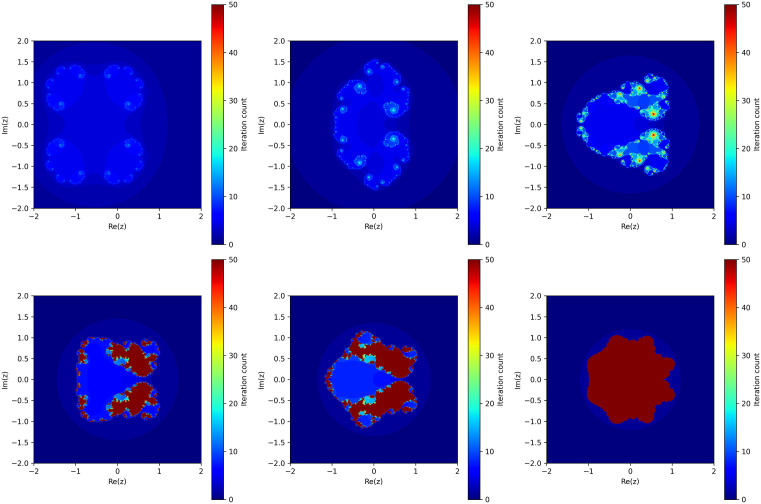
Depict the behavior of Biomorph by using the viscosity approximation method.

Fig 32 shows the behavior of Biomorph by using the viscosity approximation method with variation of the parameter *n*. This difference in *n* also greatly influences the visual composition and complexity of the biomorphs especially with respect to their symmetry, complexity, and appearance. The biomorphs have less complex more symmetrical forms at lower values of *n.* The biomorphs begin to display even more detailed information as *n* grows bigger.

**Fig 33:**
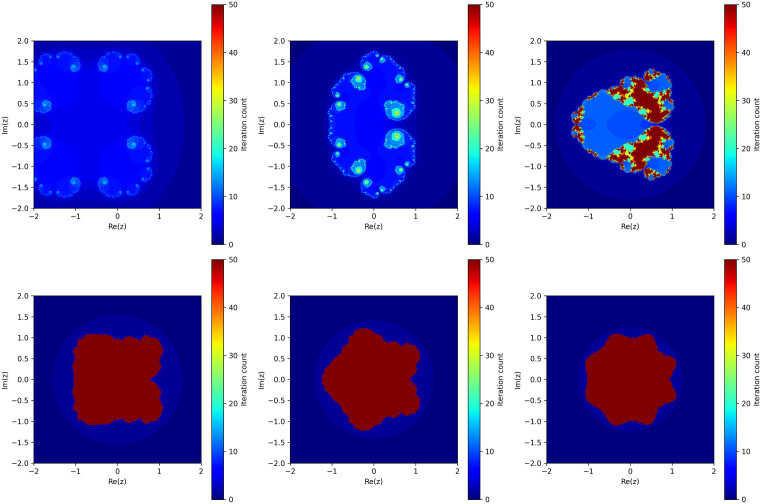
Shows the behavior of Biomorph by using the viscosity approximation process with m-convexity. The value of *m* is 0.7.

Fig 33 shows the behavior of Biomorph by using the viscosity approximation method with *m*-convexity. In which the parameter *n* is varies and other all parameters are constant. When the value of the parameter *n* increase the biomorph is be a filled and change into circular shape.

**Fig 34:**
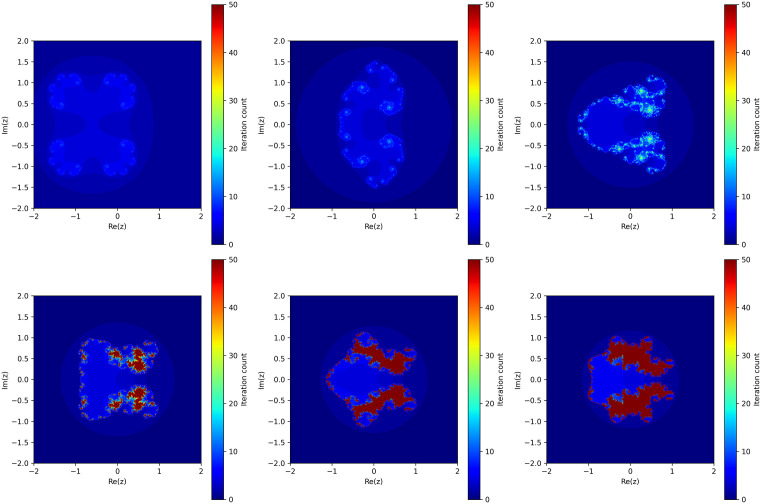
Shows the behavior of Biomorph by using the viscosity approximation process with s-convexity. The value of *s* is 0.6.

In the above figures (Fig 32–33, and Fig 34) only variation across the six subplots is the degree of the complex polynomial *n* which varies from 2 to 9 while other parameters are constant. The values of all the parameters are shown in Table 11.

**Table 11:** Variation of the parameter n.

*n*	*u*	*a*	*r*	*b*	*α*
2	0.5	0.9	0.3	0.01	0.4
3	0.5	0.9	0.3	0.01	0.4
4	0.5	0.9	0.3	0.01	0.4
5	0.5	0.9	0.3	0.01	0.4
6	0.5	0.9	0.3	0.01	0.4
9	0.5	0.9	0.3	0.01	0.4

## 7. Numerical example

In this section, we investigated the large array of shapes and sizes that Julia set, Mandelbrot sets, and Biomorphs can assume. This section is an extension of that exploration to examine the effect of the parameter a∈(0, 1) on the Julia set, Mandelbrot sets, and Biomorphs. The dependence of parameters of some iteration processes on these fractals is quite interesting. For example, Kumari et al. [18] showed that the viscosity iteration method can be used to uncover the fractal property of such sets. As a further study on these relationships, we introduce two important numerical measures: Average Escape Time (AET) and Non-Escaping Area Index (NAI), which were given in [19]. AET is calculated by computing an average of how many iterations does it take points to escape to infinity and can tell us how fast points move away from the fractal. NAI on the other hand is found by determining the ratio of points not escaping over the total points in the region of interest. This index basically refers to the portion of the area that is covered with the Mandelbrot or Julia set, which helps us to know the relative size of the fractal on the given area. To examine the effects of the value of a∈(0, 1) on Julia sets for various values of a∈(0, 1) and calculate the respective AET and NAI for each and plot them. Specifically, we ensure that we create 100 equally spaced values of in the range [−2, 2] for the Julia set. These images are produced using a resolution of 800 x 800 pixels so the fractal structures are extremely high in detail. The software used to implement the escape-time algorithms used for generating the sets were implemented in MATLAB R2024a.

### 7.1. Time and ANI plots for Julia sets

**Fig 35:**
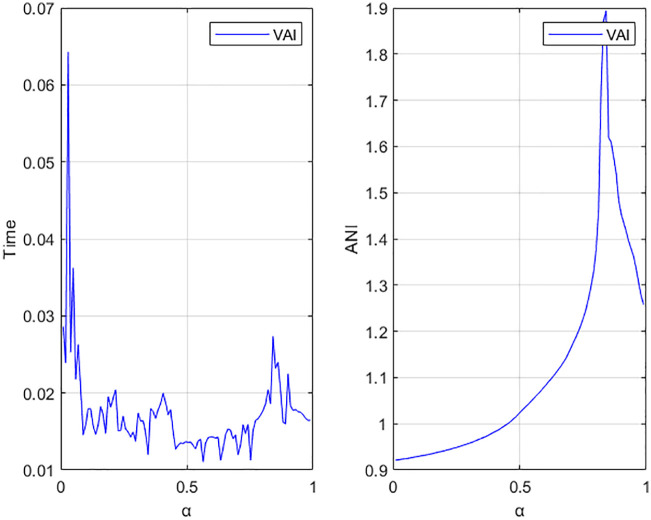
Time and ANI plots for Julia sets by using viscosity approximation method with different values of the parameter *α.*

**Fig 36:**
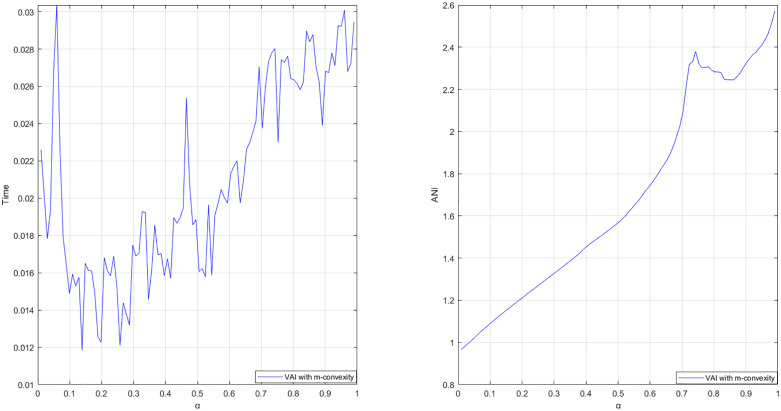
Time and ANI plots for Julia sets for viscosity approximation method with *m*-convexity by different values of the parameter *α.*

**Fig 37:**
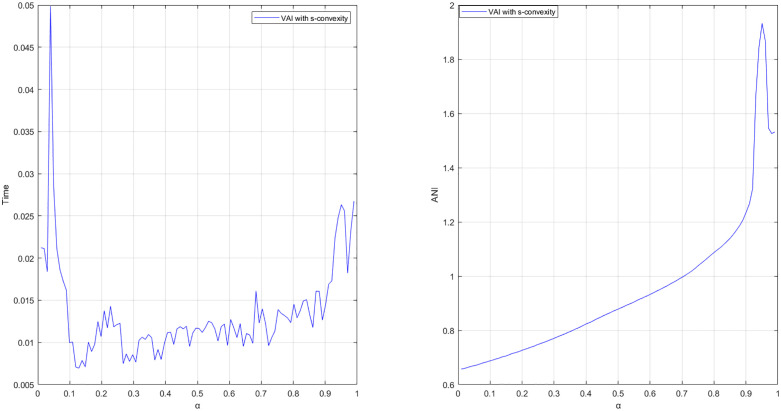
Time and ANI plots for Julia sets for viscosity approximation method with *s*-convexity by different values of the parameter *α.*

In this section, we discuss the Time and ANI Julia set for viscosity approximation method, viscosity approximation method with *m*-convexity, and viscosity approximation method with *s*-convexity for different values of *α*. In Fig 34, Fig 35, and Fig 36, we use all those values of the other parameters, that are n=4, u=0.1, r=0.5, a=0.9, and b=0.5, used in Fig 1. Fig 35, shown Time and ANI plot for Julia set with viscosity approximation method. Fig 36, is for viscosity approximation process with *m*-convexity, where the value of the parameter *m* is 0.5 while other parameters are n=4, u=0.1, r=0.5, a=0.9, and b=0.5. Fig 37 is for viscosity approximation process with *s*-convexity, where the value of the parameter *s* is 0.5 while other parameters are n=4, u=0.1, r=0.5, a=0.9, and b=0.5.

### 7.2. Mandelbrot sets

**Fig 38:**
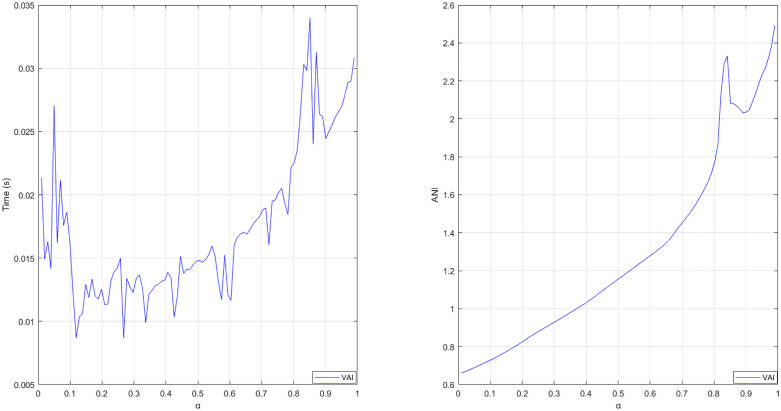
Time and ANI plots for Mandelbrot sets for viscosity approximation method with different values of the parameter *α.*

**Fig 39:**
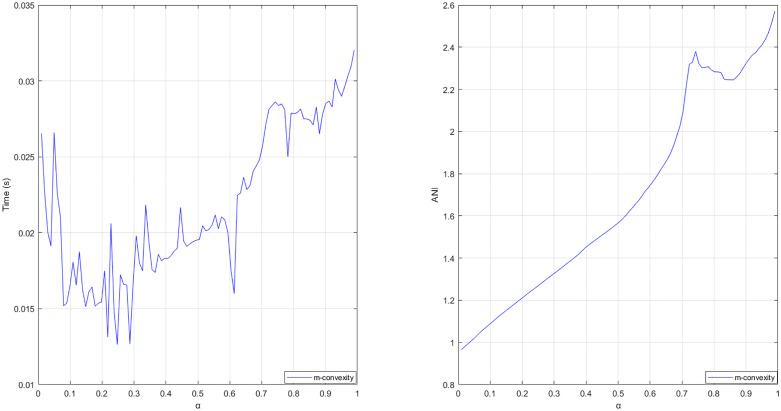
Time and ANI plots for Mandelbrot sets for viscosity approximation method with *m*-convexity by different values of the parameter *α.*

**Fig 40:**
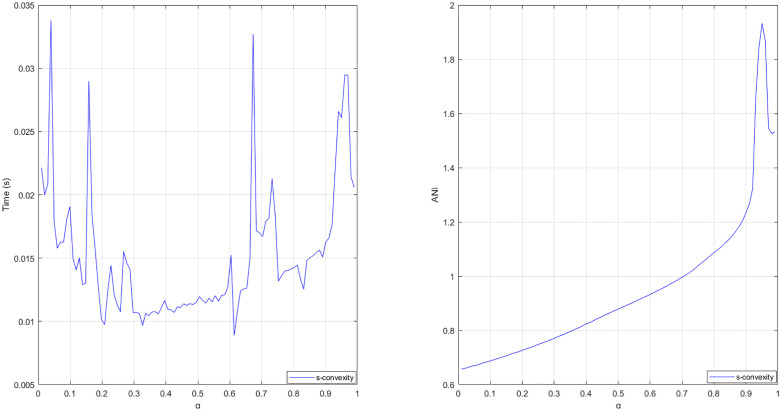
Time and ANI plots for Mandelbrot sets for viscosity approximation method with *s*-convexity by different values of the parameter *α.*

In this section, we deliberate the Time and ANI for Mandelbrot set for viscosity approximation method, viscosity approximation method with *m*-convexity, and viscosity approximation method with *s*-convexity for different values of *α*. In Fig 38, Fig 39, and Fig 40, we use all those values of the other parameters, that are used in Fig 13. Fig 38, shown Time and ANI plot for Julia set with viscosity approximation method. Fig 39, is for viscosity approximation process with *m*-convexity, where the value of the parameter *m* is 0.5. Fig 40 is for viscosity approximation process with *s*-convexity, where the value of the parameter *s* is 0.5.

### 7.3. Biomorphs

**Fig 41:**
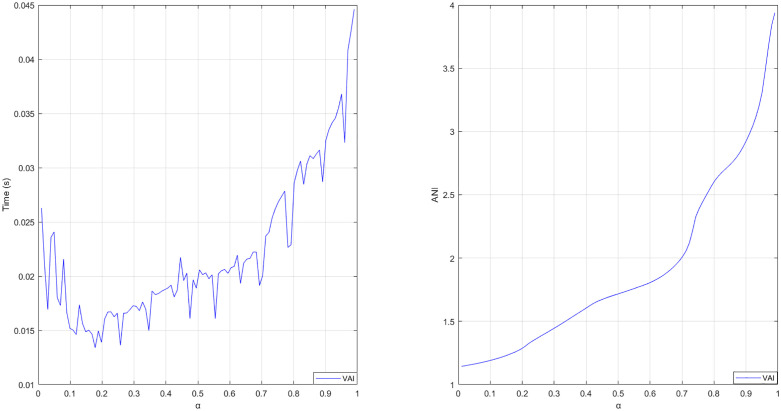
Time and ANI plots for Biomorphs for viscosity approximation method with different values of the parameter *α.*

**Fig 42:**
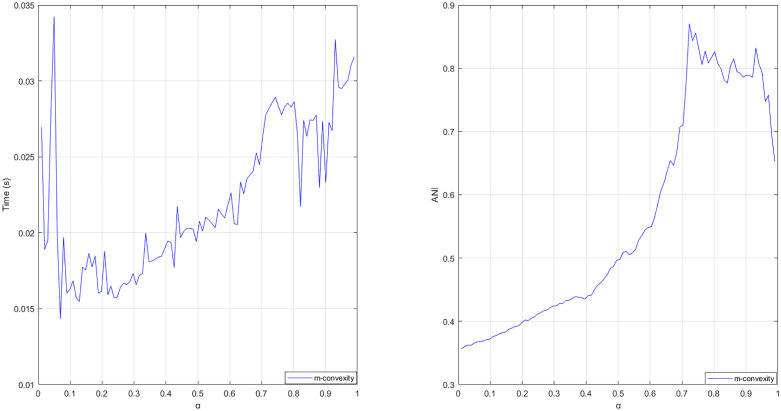
Time and ANI plots for Biomorphs for viscosity approximation method with *m*-convexity by different values of the parameter *α.*

**Fig 43:**
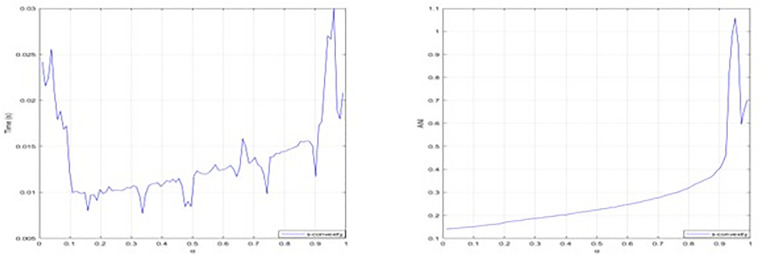
Time and ANI plots for Biomorphs for viscosity approximation method with *s*-convexity by different values of the parameter *α.*

In this section, we discuss the Time and ANI Biomorphs for viscosity approximation method, viscosity approximation method with *m*-convexity, and viscosity approximation method with *s*-convexity for different values of *α*. In Fig 41, Fig 42, and Fig 43, we use the values of parameters, are n=4, u=0.1, r=0.5, a=0.9, and b=0.5. Fig 41, shown Time and ANI plot for Julia set with viscosity approximation method. Fig 42, is for viscosity approximation process with *m*-convexity, where the value of the parameter *m* is 0.5. Fig 43 is for viscosity approximation process with *s*-convexity, where the value of the parameter *s* is 0.5.

### 7.4. Numerical results via 3D plots

In this subsection, we discuss the average escape time (AET), non-escaping area index (NAI) and time plots of Julia set, Mandelbrot set, and Biomorphs.

**Fig 44:**
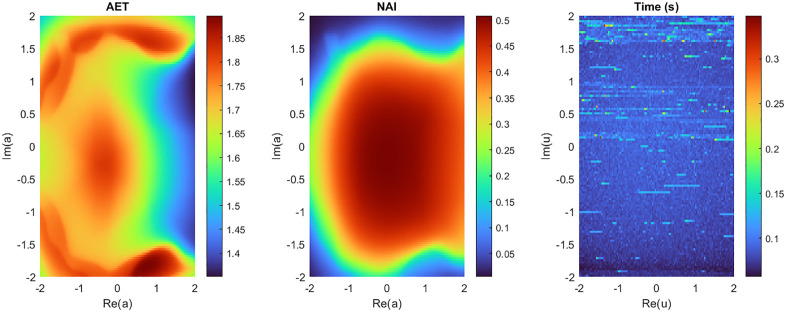
AET, NAI, and time (s) plots for Julia sets by using viscosity approximation iteration process.

**Fig 45:**
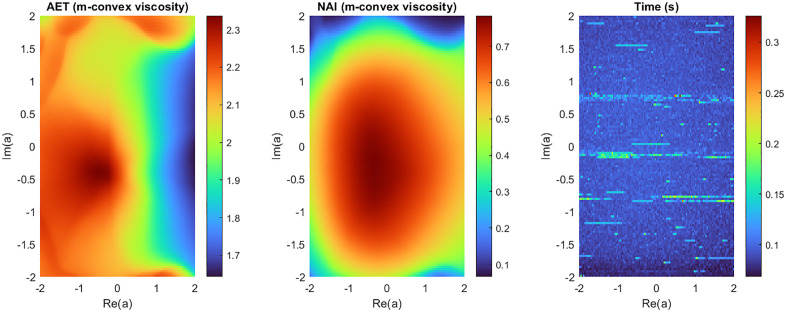
AET, NAI, and time (s) plots for Julia sets by using viscosity approximation iteration process with *m*-convexity.

**Fig 46:**
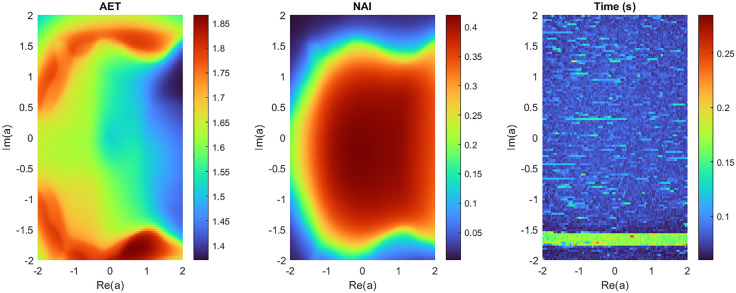
AET, NAI, and time (s) plots for Julia sets by using viscosity approximation iteration process with *s*-convexity.

Figs 44−46, shows the AET, NAI and time (in seconds) for Julia set by using viscosity approximation method, viscosity approximation method with *m*-convexity, and viscosity approximation method with *s*-convexity. Analyzing several aspects, the overall nature of AET plots seems rather similar, and only slight differences can be identified. The AET graphs indicate that the lower the points in the parameter space, the large the values of AET, and the mean velocity of escape is average. NIA plot has also the same shape as the AET plot but it is more regular than AET plot. One can see a homogeneous area on the right and upper left corners, the NAI of which is zero and brings to mind the lack of non-escaping points. The time plots do have observable noise in them because of the process and functions that underlie them.

**Fig 47:**
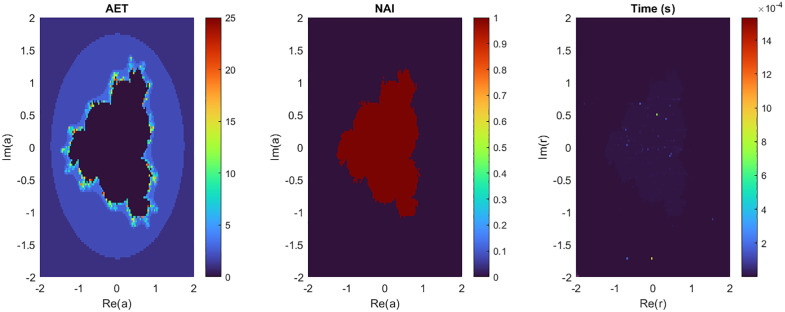
AET, NAI, and time (s) plots for Mandelbrot sets by using viscosity approximation iteration process.

**Fig 48:**
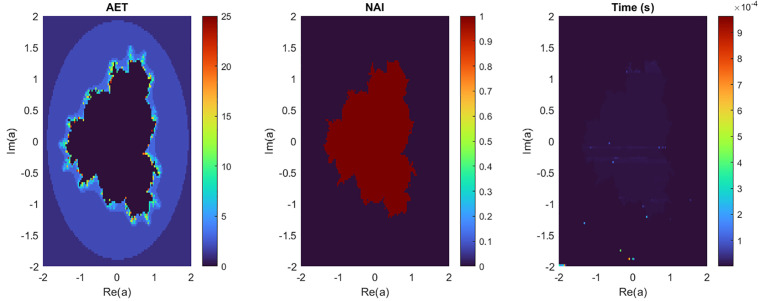
AET, NAI, and time (s) plots for Mandelbrot sets by using viscosity approximation iteration process with *m*-convexity.

**Fig 49:**
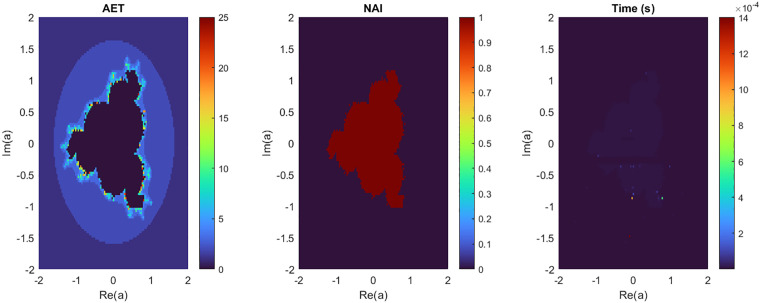
AET, NAI, and time (s) plots for Mandelbrot sets by using viscosity approximation iteration process with *s*-convexity.

Figs 47−49, shows the AET, NAI and time (in seconds) for Mandelbrot set by using viscosity approximation method, viscosity approximation method with *m*-convexity, and viscosity approximation method with *s*-convexity. Analyzing several aspects, the overall nature of AET plots seems rather similar, and only slight differences can be identified. The AET graphs indicate that the lower the points in the parameter space, the large the values of AET, and the mean velocity of escape is average. NIA plot has also the same shape as the AET plot but it is more regular than AET plot. One can see a homogeneous area on the right and upper left corners, the NAI of which is zero and brings to mind the lack of non-escaping points. The time plots do have observable noise in them because of the process and functions that underlie them.

**Fig 50:**
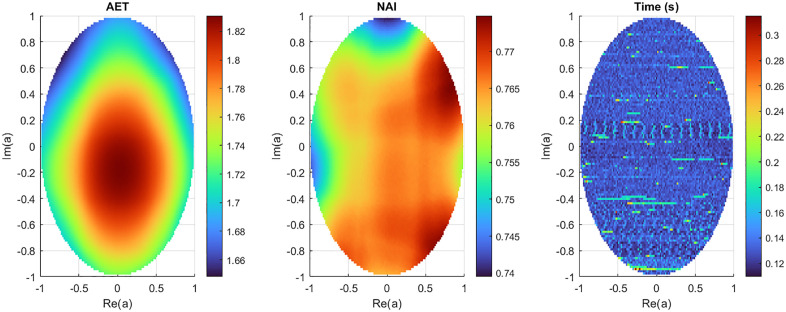
AET, NAI, and time (s) plots for Biomorphs by using viscosity approximation iteration process.

**Fig 51:**
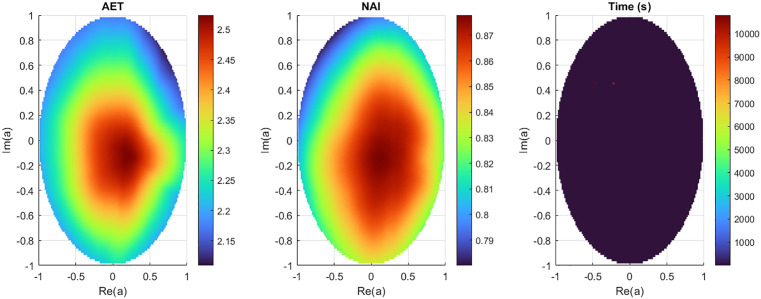
AET, NAI, and time (s) plots for Biomorphs by using viscosity approximation iteration process with *m*-convexity.

**Fig 52:**
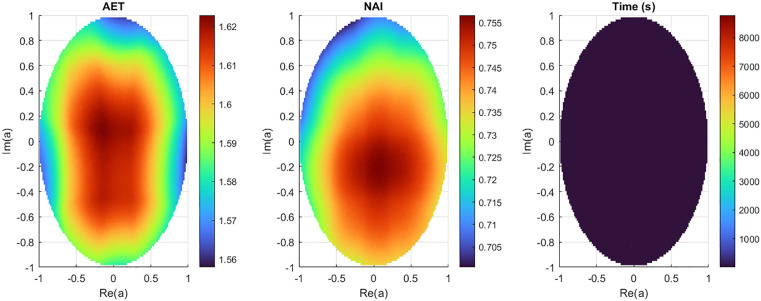
AET, NAI, and time (s) plots for Biomorphs by using viscosity approximation iteration process with *s*-convexity.

Figs 50−52, shows the AET, NAI and time (in seconds) for Biomorphs by using viscosity approximation method, viscosity approximation method with *m*-convexity, and viscosity approximation method with *s*-convexity. Analyzing several aspects, the overall nature of AET plots seems rather similar, and only slight differences can be identified. The AET graphs indicate that the lower the points in the parameter space, the large the values of AET, and the mean velocity of escape is average. NIA plot has also the same shape as the AET plot but it is more regular than AET plot. One can see a homogeneous area on the right and upper left corners, the NAI of which is zero and brings to mind the lack of non-escaping points. The time plots do have observable noise in them because of the process and functions that underlie them.

## 8. Comparison

In this section, we discuss the comparison between viscosity approximation iteration, viscosity approximation iteration process with *m*-convexity, and viscosity approximation iteration process with s-convexity.

**Table:** 

Feature	Viscosity Approximation iteration	Viscosity Approximation iteration with *m*-Convexity	Viscosity Approximation iteration with *s*-Convexity
Convexity Type	Classical convexity	m-convexity	s-convexity
Control Parameters	α∈(0, 1)	α∈(0, 1), m∈[0, 1]	α, s ∈ (0,1)
Iteration Flexibility	Low	Moderate	High
Escape Speed	Moderate	Faster than Viscosity Approximation iteration	Fastest
Boundary Sharpness	Smooth boundaries	Sharper boundaries	Finest and sharpest
Fractal Complexity	Low to moderate	High	Very high
Symmetry Breaking	Weak	Moderate	Strong
Numerical Stability	High	High	Medium
Visual Richness	Basic	Enhanced	Maximum

## 9. Conclusion

In this manuscript, we presented a complete iterative framework for the generation of Mandelbrot sets, Julia sets and Biomorph by using viscosity approximation technique for a complex function $W(z)=z^{n}+uz+r,$ where n≥2 and u,r∈C. Based on fixed-point theory, this generalized scheme generates strong convergence properties we develop an escape-time criterion of this generalized iteration process. We generalized the viscosity approximation process with m-convexity and m-convexity. Variations in important parameters including the degree of the polynomial, viscosity coefficient, and affine transformation constants produce an amazing Julia, Mandelbrot set fractals and Biomorph. We use MATLAB R2024a to generate visually complex and high-resolution fractals. Acknowledging the limitations of this study, we note that the iterative schemes and escape criteria are specifically tailored to the polynomial $W(z)=z^{n}+uz+r$ and may not directly apply to other complex functions. The computational demands, while acceptable for offline fractal generation, limit real-time interactivity. Nevertheless, the strengths of our framework lie in its rigorous theoretical foundation, the comparative visual and quantitative analysis of three convexity variants, and the clear demonstration of how *m*- and *s*-convexity refine fractal boundaries and increase complexity. These results provide a solid basis for future extensions to higher-dimensional complex spaces and for the development of more efficient algorithms. In future, we should be applying the proposed method in quaternionic or bi-complex domains as well as in higher-dimensional complex spaces.
